# Stitching together Multiple Data Dimensions Reveals Interacting Metabolomic and Transcriptomic Networks That Modulate Cell Regulation

**DOI:** 10.1371/journal.pbio.1001301

**Published:** 2012-04-03

**Authors:** Jun Zhu, Pavel Sova, Qiuwei Xu, Kenneth M. Dombek, Ethan Y. Xu, Heather Vu, Zhidong Tu, Rachel B. Brem, Roger E. Bumgarner, Eric E. Schadt

**Affiliations:** 1 Sage Bionetworks, Seattle, Washington, United States of America; 2 Department of Microbiology, University of Washington, Seattle Washington, United States of America; 3 Safety Assessment, Merck & Co., Inc., West Point, Pennsylvania, United States of America; 4 Molecular Profiling, Merck Research Laboratories, Boston, Massachusetts, United States of America; 5 Department of Molecular and Cell Biology, University of California at Berkeley, Berkeley, California, United States of America; 6 Department of Genetics and Genomic Sciences, Mount Sinai School of Medicine, New York City, New York, United States of America; Johns Hopkins University, United States of America

## Abstract

Cells employ multiple levels of regulation, including transcriptional and translational regulation, that drive core biological processes and enable cells to respond to genetic and environmental changes. Small-molecule metabolites are one category of critical cellular intermediates that can influence as well as be a target of cellular regulations. Because metabolites represent the direct output of protein-mediated cellular processes, endogenous metabolite concentrations can closely reflect cellular physiological states, especially when integrated with other molecular-profiling data. Here we develop and apply a network reconstruction approach that simultaneously integrates six different types of data: endogenous metabolite concentration, RNA expression, DNA variation, DNA–protein binding, protein–metabolite interaction, and protein–protein interaction data, to construct probabilistic causal networks that elucidate the complexity of cell regulation in a segregating yeast population. Because many of the metabolites are found to be under strong genetic control, we were able to employ a causal regulator detection algorithm to identify causal regulators of the resulting network that elucidated the mechanisms by which variations in their sequence affect gene expression and metabolite concentrations. We examined all four expression quantitative trait loci (eQTL) hot spots with colocalized metabolite QTLs, two of which recapitulated known biological processes, while the other two elucidated novel putative biological mechanisms for the eQTL hot spots.

## Abstract 2

DNA variation can be used as a systematic source of perturbation in segregating populations as a way to infer regulatory networks via the integration of large-scale, high-dimensional molecular profiling data.

## Author Summary

It is now possible to score variations in DNA across whole genomes, RNA levels and alternative isoforms, metabolite levels, protein levels and protein state information, protein–protein interactions, and protein–DNA interactions, in a comprehensive fashion in populations of individuals. Interactions among these molecular entities define the complex web of biological processes that give rise to all higher order phenotypes, including disease. The development of analytical approaches that simultaneously integrate different dimensions of data is essential if we are to extract the meaning from large-scale data to elucidate the complexity of living systems. Here, we use a novel Bayesian network reconstruction algorithm that simultaneously integrates DNA variation, RNA levels, metabolite levels, protein–protein interaction data, protein–DNA binding data, and protein–small-molecule interaction data to construct molecular networks in yeast. We demonstrate that these networks can be used to infer causal relationships among genes, enabling the identification of novel genes that modulate cellular regulation. We show that our network predictions either recapitulate known biology or can be prospectively validated, demonstrating a high degree of accuracy in the predicted network.

## Introduction

Cells are complex molecular machines that employ multiple levels of regulation that enable them to respond to genetic and environmental perturbations. Advances in biology over the past several years to elucidate the complexity of this regulation have been truly astonishing. However, despite transformative advances in technology, it remains difficult to assess where we are in our understanding of cell regulation, relative to a complete comprehension of such a process. One of the primary difficulties in our making such an assessment is that the suite of research tools available to us seldom provides insights into aspects of the overall picture of the system that are not directly measured. While different technologies provide information that our analytical tools, both algorithmic and intellectual, seek to combine into a coherent picture, one of the primary limitations of the majority of analytical tools in use today is a focus on single dimensions of data, rather than on maximally integrating data across many different dimensions simultaneously to view processes more completely, thereby achieving a greater understanding of these processes.

The full suite of interacting parts in a cell over time, if they could be viewed collectively, would enable our achieving a more complete understanding of cellular processes, much in the same way we achieve understanding by watching a movie. The continuous flow of information in a movie enables our minds to exercise an array of priors that provide context and constrain the possible relationships (structures), while our internal network reconstruction engine pieces all of the information together regarding the highly complex and nonlinear relationships represented in the movie, so that in the end we are able to achieve an understanding of what is depicted at a hierarchy of levels. If instead of viewing a movie as a continuous stream of frames of coherent pixels and sound, we viewed single dimensions of the information independently, understanding would be difficult if not impossible to achieve. For example, consider viewing a movie as independent, one dimensional slices through the frames of the movie, where each slice is viewed as pixel intensities across that one dimension changing over time (like a dynamic mass spec trace). In this way it would be very difficult to understand the meaning of the movie by looking at all of the one dimensional traces independently.

Despite the complexity of biological systems, even at the cellular level, research in the context of large-scale high dimensional -omics data has tended to focus on single data dimensions, whether constructing coexpression networks on the basis of gene expression data, carrying out genome-wide association analyses on the basis of DNA variation information, or constructing protein interaction networks on the basis of protein–protein interaction data. While we achieve some understanding in this way, progress is limited because none of the dimensions on their own provide a complete enough context within which to interpret results fully. This type of limitation has become apparent in genome-wide association studies (GWAS), where many hundreds of highly replicated loci have been identified and highly replicated as associated with disease; but our understanding of disease is still limited because the genetic loci do not necessarily inform on the gene affected, on how gene function is altered, or more generally, how the biological processes involving a given gene are altered [1]–[4]. It is apparent that if different biological data dimensions could be formally considered simultaneously, we would achieve a more complete understanding of biological systems [2],[3],[5]–[7]. (See the documentary film *The New Biology* at http://www.youtube.com/watch?v=sjTQD6E3lH4.)

Therefore, to form a more complete understanding of biological systems, we must not only evolve technologies to sample systems at ever higher rates and with ever greater breadth, we must innovate methods that consider many different dimensions of information to produce more descriptive models (movies) of the system. Methods are emerging that integrate pairs of data dimensions. For example, we recently developed methods that simultaneously integrate DNA variation and RNA expression data generated in a population context to identify coherent modules of interconnected gene expression traits driven by common genetic factors [2],[8]. In addition, many groups have begun incorporating a time dimension in the context of high-dimensional molecular-profiling data to elucidate how networks can transform over time [9],[10].

**Figure 1:**
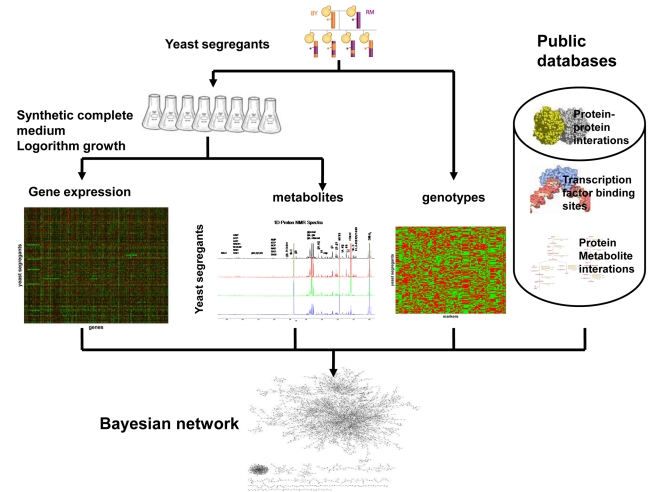
Overview of the experimental design. A cross between laboratory (BY) and wild (RM) strains of *S. cerevisiae* [11] was gene expression profiled. Metabolites were profiled under the same conditions. These data were then integrated with genotype data along with information from public databases to derive a BN. The derived network was used to analyze how cells are regulated.

Here we develop and apply a network reconstruction approach that simultaneously integrates six different types of data: endogenous metabolite concentration, RNA expression, DNA variation, DNA–protein binding, protein–metabolite interaction, and protein–protein interaction data, to construct probabilistic causal networks that elucidate the complexity of cell regulation (Figure 1). The goals of our integrative analysis are not only to find causal regulators underlying expression quantitative trait loci (eQTL) hot spots, but to uncover mechanisms by which these predicted causal regulators affect genes and metabolites whose transcriptional profiles or metabolite profiles are linked to the eQTL hot spots. We leveraged a previously described cross between laboratory (BY) and wild (RM) yeast strains (referred to here as the BXR cross) for which DNA variation and RNA expression had been assessed [11],[12], to carry out a quantitative metabolite profiling using quantitative NMR (qNMR) under the same experimental conditions as the gene expression study [12]–[14]. We demonstrate that, like transcript and protein levels, concentrations of many metabolites are strongly linked to metabolite QTLs (metQTLs). Several of the metQTLs are seen to colocalize with expression quantitative trait loci (eQTLs) previously identified in the same yeast population [13], enabling us to infer causal relationships between metabolites and expression traits [13],[14]. Then, by extending a previously described Bayesian network (BN) reconstruction algorithm [13], we constructed a probabilistic causal network by integrating metabolite levels, genotype, gene expression, transcription factor (TF) binding, and protein–protein interaction data. The resulting network not only validates the functional importance of eQTL hot spots in the BXR cross, but elucidates the mechanisms by which variation in DNA at eQTL hot spots affect gene expression. By systematically using the networks to elucidate the regulators of these eQTL hot spots, we are not only able to recapitulate known regulatory mechanisms, we are able to provide a number of novel and experimentally supported causal relationships predicted by our network, including that cellular amino acid concentrations are related to both amino acid biosynthesis pathways and amino acid degradation pathways, with *VPS9* predicted and prospectively validated as a key driver of a previously identified eQTL hot spot that could not previously be well characterized. In addition, we further experimentally demonstrated that *PHM7*, a previously predicted and validated causal regulator for stress response genes whose expression variations are linked to the *PHM7* locus on Chromosome XV, affected trehalose, a yeast metabolite product of the stress response pathway. These results combined not only help uncover the mechanisms by which gene expression profiles are regulated by metabolite profiles, but they also confirm the importance of gene expression in understanding system-wide variation linked to genetic perturbations.

## Results

### Characterizing Metabolite Levels in a Segregating Yeast Population

#### Experimental context matters for inferring causal relationships

Two classes of data were employed to reconstruct probabilistic causal networks: (1) DNA variation, gene expression, and metabolite data measured in the BXR cross (referred to here as BXR data), and (2) protein–DNA binding, protein–protein interaction, and metabolite–protein interaction data available from public data sources and generated independently of the BXR cross (referred to here as non-BXR data). The BXR data are reflected as nodes in the network, where edges in the network reflect statistically inferred causal relationships among the expression and metabolite traits (Methods) [13]. The non-BXR interaction data from public sources are used to derive structure priors on the network to both constrain the size of the search space in finding the best network and enhance the ability to infer causal relationships between the network nodes [13].

The BXR data in particular, directly representing the nodes and associations in the network, require special consideration given that relationships among genes and between genes and metabolites may be condition specific, requiring that the expression and metabolite data be generated under identical experimental conditions to maximize the power to identify causal relationships. In fact, others have shown that there are widespread interactions between genetic and environmental factors [15]. Just as genetic factors may predispose some populations to certain human diseases, environmental factors like diet can also increase or decrease the risk of disease [16]–[18]. Both F2 mouse [19] and rat [20] studies demonstrate that cholesterol QTLs are dependent on diet, and similarly for obesity-related traits [21],[22].

Therefore, before profiling metabolite levels in the BXR cross, we explored the importance of context in identifying associations between different molecular phenotypes by examining the expression profiles of the yeast segregants in this cross and corresponding QTLs under glucose and ethanol growth conditions [23]. Genetic variations (such as SNPs) give rise to variations in phenotypes, including quantitative traits such as gene expression and clinical traits [13],[14],[24]. Cis-acting (or proximal) eQTLs are special because they represent associations between DNA variation at a given locus where the corresponding gene physically resides and the expression levels of the corresponding gene, reflecting in most causes allelic differences in transcript levels [13],[24],[25]. For the yeast segregants comprising the BXR cross, expression data have been generated under glucose and ethanol growth conditions [23]. For both expression sets the underlying genetic perturbations in the BXR cross are identical. We identified 548 and 569 cis-eQTLs for the glucose and ethanol data, respectively, at the *p*-value cutoff where less than 1 false positive is expected genome wide. However, when the two sets of cis-eQTLs were compared, we found that only two-thirds of the cis-eQTLs were common, where half of the total cis-eQTLs were unique to one of the two conditions (Figure S1a). It is worth noting that for cis-eQTL detected in one condition, the corresponding LOD scores in the other condition are approximately uniformly distributed over the entire LOD score range (Figure S1b and S1c). This result suggests that nonoverlapping cis-eQTLs across the different conditions are not due to a lack of power to replicate a given eQTL in the second condition, but to condition-specific effects (medium conditions in this instance).

Even under highly similar growth conditions, we can see that slight differences in amino acid concentrations lead to significant changes in gene expression. For example, in the original screen of the BXR cross [11], there were 203 gene expression traits linked to the *LEU2* locus at a 5% false discovery rate (FDR) (in this instance the leucine concentration in the medium was 80 mg/l). In a follow-up screen of this same cross [23], the leucine concentration in an otherwise identical growth medium was 100 mg/l; only 57 genes with expression levels linked to the *LEU2* locus were detected in this case at a 5% FDR. Greater than 60% (36) of these 57 genes overlapped the set of 203 genes, a very significant overlap (*p* = 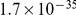) indicating common biological processes were affected (Table S1). The smaller number of genes linked to the *LEU2* locus in the follow up dataset is consistent with the known relationship between leucine concentrations in growth medium and leucine biosynthesis: high levels of leucine in growth medium represses leucine biosynthesis [26]. This result implies that considerable differences may exist in the regulatory networks between different growth conditions. Therefore, to reliably infer causal relationships between variations in gene expression and metabolite levels, it is critical to measure them under identical conditions.

#### Measuring metabolite levels in the BXR cross

To maximize the power to detect relationships between metabolite and gene expression traits in the BXR cross, we measured metabolite levels in cell extracts from the 120 yeast segregants composing the cross after culturing the segregants using growth conditions that were identical to the growth conditions used to generate the gene expression data (Methods). Cellular metabolite concentrations can be measured by mass spectrometry (MS) [27] technologies or qNMR [28],[29]. While MS technologies are more sensitive and can detect low-abundance metabolites, accurate quantification requires the addition of an internal standard for each metabolite to be measured. This can be accomplished by generating isotope-enriched metabolite extracts (repetitively growing cells in medium containing isotope-enriched nutrients such as ^13^C_6_-glucose) [27]. Quantitative LCXMS/MS analyses are then based on isotope ratios constructed by adding unlabeled endogenous metabolites of known amounts as internal controls, enabling an accurate quantification of metabolite levels [27],[30]. In contrast to MS-based metabolite profiling, qNMR requires no special sample preparation procedures, although it is less sensitive than MS techniques. With qNMR, a single internal reference standard in an NMR sample is sufficient to quantify all detectable endogenous metabolites. Therefore, we generated metabolite profiling data using qNMR in the BXR cross under the same growth condition as the gene expression profiles were generated (Figure 1).

#### Metabolite abundances are under genetic control

Each cell extract was analyzed on a 700-MHz NMR spectrometer by acquiring one-dimensional proton spectra. The appearance and density of peaks in these scans (Figure S2) were in keeping with previously reported yeast NMR spectra [31]. Metabolites were identified on the basis of NMR reference spectra encompassing more than 700 endogenous compounds. Quantities of metabolites in each sample were calculated on the basis of integrated peak areas with respect to the concentration of the internal reference standard DSS-*d_6_*. From the NMR reference spectra, we were able to identify and accurately quantify 56 yeast endogenous metabolites excluding ethanol and methanol (see Methods). We reported the amount of each metabolite as nanomoles per yeast cell.

The averaged metabolite concentrations for all segregants are listed in Table S2. Concentrations of a number of amino acids, including lysine, glycine, and isoleucine, were consistent with previous measurements of intracellular concentrations [32],[33]. The average intracellular concentration of leucine was lower than previously reported values [32],[33], an expected result if synthetic media were not a fully sufficient source of this amino acid. Such activity would be consistent with reports of the activity of the leucine biosynthesis pathway from gene expression [12] and proteomic [34] measurements of yeast grown under similar conditions. Arginine, AMP, ADP, and ATP concentrations were also low relative to previous measurements [32],[33], but the average energy charge was 0.833, and the average NAD/NADH ratio is 20.022, both within expected normal ranges [35],[36].

To assess the variation of metabolite concentrations across biological replicates of independent yeast cultures, we collected ten replicate cell extracts for each parental strain at two time points separated by 2 mo. A comparison between the two parental strains identified 23 metabolites with significantly different concentrations (Wilcoxon test *p*<0.005) (Table S3), indicating that intersample variation for the different strains was significantly smaller than interstrain variation for these metabolites.

**Figure 2:**
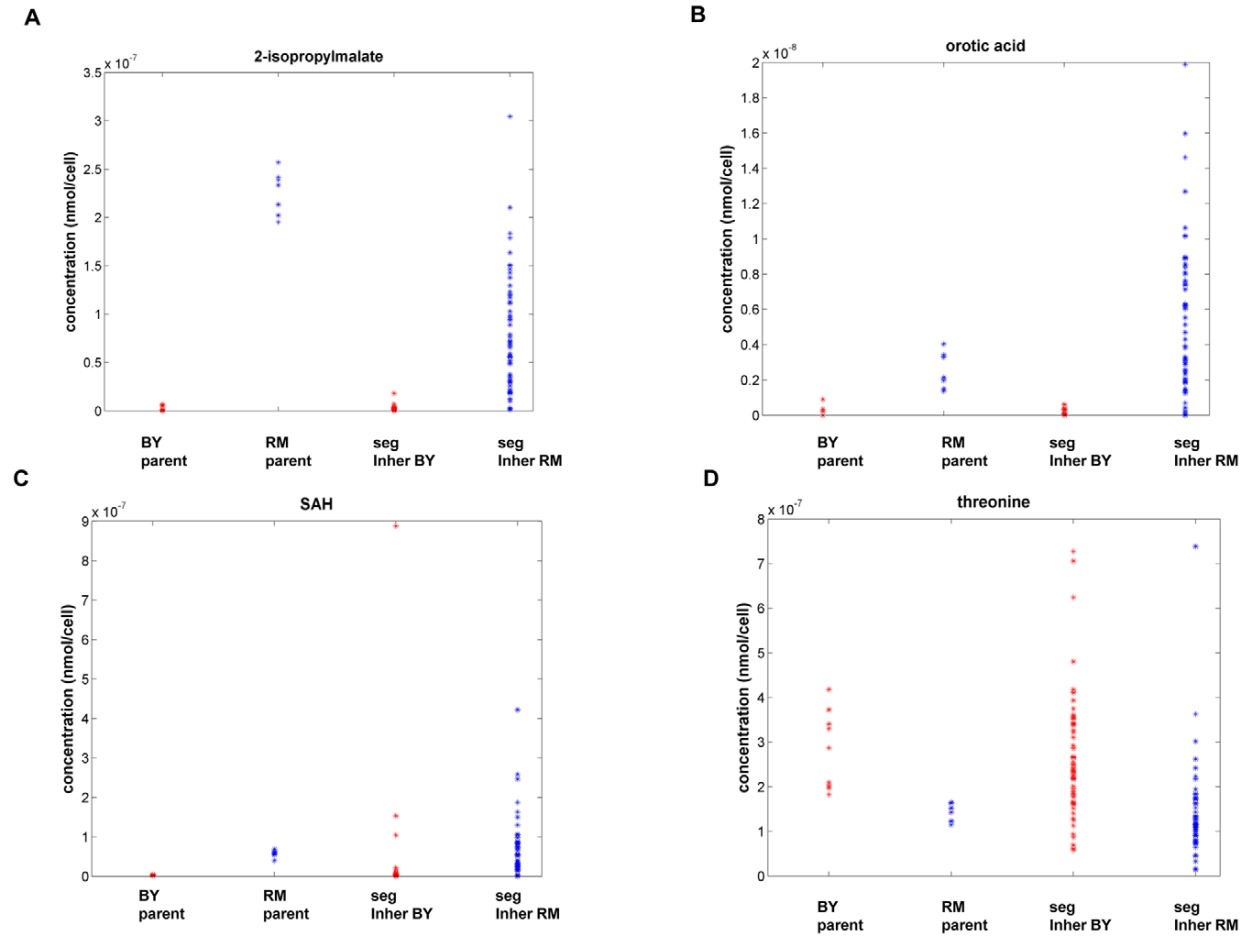
Distributions of metabolite concentrations between parental strains and among 120 segregants of a cross between laboratory (BY) and wild (RM) strains of *S. cerevisiae* [11]. The *y*-axis is metabolite concentrations (nanomoles per yeast cell). The genotypes for segregants are reported at the loci to which the metabolite concentrations were linked. Represented are the metabolites (A) 2-isopropylmalate; (B) orotic acid; (C) SAH; and (D) threonine.

We next sought to identify genetic loci that were segregating in the BXR cross with metabolite levels. We used more than 2,000 SNP markers uniformly spaced throughout the yeast genome [11] to map genetic loci for the 56 reliably scored metabolites. The peak LOD scores for 16 metabolites (29%) exceeded the genome-wide significance LOD score threshold of 3.9 (FDR = 0.05) (Table 1). This percentage is similar to the percentage of gene expression traits that give rise to a similarly significant linkage signal. Eleven of the 16 metabolite traits with significant metQTLs (Table 1) were also found to have significantly different concentrations in the parental strains (Figure 2). We examined these metQTL results in the context of QTLs controlling gene expression (eQTL) previously detected in the same BXR cross [24]. Twelve of the 16 metQTLs were coincident with four previously identified eQTL hot spot regions to which many more gene expression traits linked in trans than would be expected by chance [13],[24]. Phenylpyruvate, 2-isopropylmalate, alanine, arginine, and NAc-glutamate levels were linked to position 100,000 bp on Chromosome III, which was associated with eQTL hot spot 1; orotic acid and dihydroorotic acid levels were linked to position 130,000 bp on Chromosome V, which was associated with eQTL hot spot 2; isoleucine, threonine, and valine levels were linked to position 70,000 bp on Chromosome XIII, which was associated with eQTL hot spot 3; trehalose and glycerol levels were linked to position 180,000 bp on Chromosome XV, which was associated with eQTL hot spot 4 (Table 1).

**Table 1:** Metabolite concentrations that are under significant genetic control in the BXR cross (LOD score>3.9 corresponds to FDR 0.05), where the metabolite QTL are coincident with eQTL hot spots.

Metabolite	QTL				
	metQTL			eQTL	
	Chromosome	Position	LOD Score	n Genes Linked to the Locus	eQTL Hot Spot
Phenylpyruvate	III	91287	4.05074	203	1
2-isopropylmalate^a^	III	91496	15.4214	203	1
Alanine^a^	III	76127	8.399	203	1
Arginine^a^	III	91977	5.67128	203	1
NAc-glutamate^a^	III	91977	5.86485	203	1
Orotic acid^a^	V	116812	15.4214	41	2
Dihydroorotic acid^a^	V	117705	4.47374	41	2
SAH^a^	VIII	167506	13.4212	14	NA
SAM^a^	VIII	167506	10.3425	14	NA
Isoleucine^a^	XIII	49894	11.1032	41	3
Threonine^a^	XIII	49903	10.728	41	3
Valine	XIII	46070	4.00333	41	3
Glycerol	XV	175594	4.38217	343	4
Lysine^a^	XV	59733	8.71851	343	4
Trehalose	XV	174364	6.03112	343	4
Tyrosine	XV	89229	4.48397	343	4

a Of the metabolites listed, 11 are significantly different between the BXR parental strains as well.

### Integrating Metabolite and Other -Omics Data to Construct Networks That Elucidate eQTL Hot Spots

Given the strong genetic signal detected in the metabolite data and the coincidence of metQTL and eQTL hot spot regions, we set out to explore an integrated network analysis strategy using the gene expression profiles [11] as well as the metabolite data described above. Gene expression and metabolite traits were treated equivalently as nodes in our BN reconstruction process. As such, we modified our previously reported BN reconstruction method [13] to accommodate metabolite data, in addition to genotype, gene expression, protein interaction, and TF–DNA binding data. The KEGG biochemical pathway database [37] was used to generate structure priors between metabolites and genes encoding enzymes known to be involved in biochemical reactions in canonical pathways. Intuitively, genes encoding enzymes that directly catalyze biochemical reactions for the metabolites were assigned stronger prior probabilities of being related during network reconstruction, whereas genes that encode enzymes catalyzing downstream or upstream biochemical reactions of the metabolites were assigned weaker priors (see Methods for details). Differentially regulated genes and the structure priors for genotype, TF–DNA, and protein–protein interaction data were defined as previously described [13].

The 56 reliably quantified metabolites were included as input into the BN reconstruction program. From this probabilistic causal network we can identify subnetworks for all of the metabolites or any set of genes (see Methods for details). To assess the predictive power of this network, we examined how metabolites and gene expression traits relate to one another at the four eQTL hot spots in Table 1, providing for the possibility of elucidating regulatory mechanisms and generating testable hypotheses about novel regulatory relationships.

#### Subnetwork linked to eQTL hot spot 1

**Figure 3:**
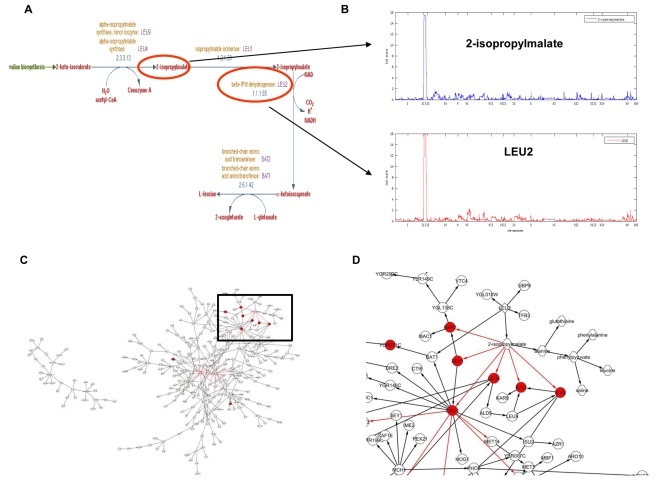
Relationship between 2-isoproplymalate and genes linked to eQTL hot spot 1 on Chromosome III. (A) 2-isopropylmalate is an intermediate metabolite in the leucine biosynthesis pathway and *LEU2* is a key enzyme in this pathway; (B) 2-isopropylmalate concentrations are linked to the *LEU2* locus and is reactive to *LEU2* expression; (C) 2-isopropylmalate is reactive to *LEU2* and causal for genes with Leu3p binding sites (red nodes); (D) a zoomed-in view of the subnetwork highlighted in (C) (around 2-isopropylmalate). Hexagon-shaped nodes represent metabolites, circular nodes represent genes, and diamond-shaped nodes represent genes with cis-eQTLs.

We [13],[24] and others [38] have previously inferred the identity of multiple causal variants affecting the expression levels of many genes at eQTL hot spot 1 (the engineered deletion at *LEU2* and natural variation at *ILV6*). We previously hypothesized that *LEU2* affected many gene expression traits linked to this hot spot by regulating genes that bind the Leu3p TF. We demonstrated that genes in the *LEU2* subnetwork and genes with Leu3p binding sites were overrepresented among the set of genes making up the *LEU2* transcriptional knockout signature [13]. However, despite the strong statistical and empirical evidence implicating *LEU3*, we found that *LEU3* expression levels did not significantly vary in the BXR cross (Figure S3), suggesting a missing link between the *LEU2* genotype and Leu3p activity resulting in widespread effects on transcription. In addition to Leu3p concentration and *LEU3* gene expression, Leu3p activity is known to be regulated by 2-isoprolylmalate, an intermediate product in leucine biosynthesis [39]. By incorporating the metabolite data into the network reconstruction procedure, we found that levels of 2-isopropylmalate were strongly linked to the *LEU2* locus, and that *LEU2* expression was strongly supported as causal for the abundance levels of 2-isopropylmalate (Figure 3B). Our integrated BN indicates that variation in levels of this metabolite are a consequence of changes in *LEU2* expression (Figure 3C and 3D), and changes in 2-isopropylmalate levels are causal for expression levels of genes with Leu3p binding sites (Figure 3C and 3D). 2-isopropylmalate is a key intermediate in the leucine biosynthesis pathway (Figure 3A), which activates Leu3p and results in upregulation of its target genes [39]. Therefore, our integrated view of the data suggests that the metabolite 2-isopropylmalate is the missing link between *LEU2* and Leu3p regulated genes. In fact, the subnetwork associated with this eQTL hot spot (Figure 3D) suggests a regulatory mechanism: 2-isopropylmalate mediates the effect of *LEU2* genotype on mRNA expression of Leu3p targets and metabolites, including alanine, glutathione, phenylpyruvate, valine, phenylananine, and leucine (Figure 3D). Such regulatory mechanism is consistent with known regulatory mechanisms of Leu3p and leucine biosynthesis.

Arginine and N-acetyl-glutamate (NAc-glutamate) are metabolites in the arginine biosynthesis pathway (Figure S4A). Variations in arginine and NAc-glutamate levels in the BXR cross were also linked to eQTL hot spot 1 (Figure S4B). The metQTLs for arginine and NAc-glutamate at this locus were close to genes encoding arginine biosynthesis enzymes and TFs in our BN (Figure S4C), consistent with the known role of NAc-glutamate as an arginine biosynthetic intermediate. In this subnetwork, transcript levels of *CPA2*, a gene involved in the biosynthesis of the arginine precursor citrulline, regulate concentrations of arginine and, further downstream, NAc-glutamate. These results combined with the inference from our network that *ARG4* is a key node in the eQTL hot spot 1 subnetwork (Figure S4C), recapitulate the known arginine biosynthesis pathway. Interestingly, we detected a negative correlation between NAc-glutamate and arginine concentrations across the panel of BXR strains, suggesting that feedback control points in this pathway lie between these two metabolites. Our network suggested that sequence variation in *ILV6* was causal for gene expression variation in *GCN4*, a master transcriptional regulator of amino acid biosynthesis genes, which in turn is causal for expression variation in TFs *RTG3* and *GLN3*, and then changes in the arginine biosynthesis subnetwork more generally in the BXR cross. Such a model is consistent with the overlaps we observed between the transcriptional profiles of the *ILV6* and *LEU2* knockouts [13] and this subnetwork (Fisher exact test *p* = 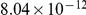 and 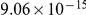, respectively). Taken together, our results indicate that the constructed network in many cases not only recapitulates known biology in general, but elucidates regulatory mechanisms, such as networks governing amino acid biosynthesis.

#### Subnetwork linked to eQTL hot spot 2

**Figure 4:**
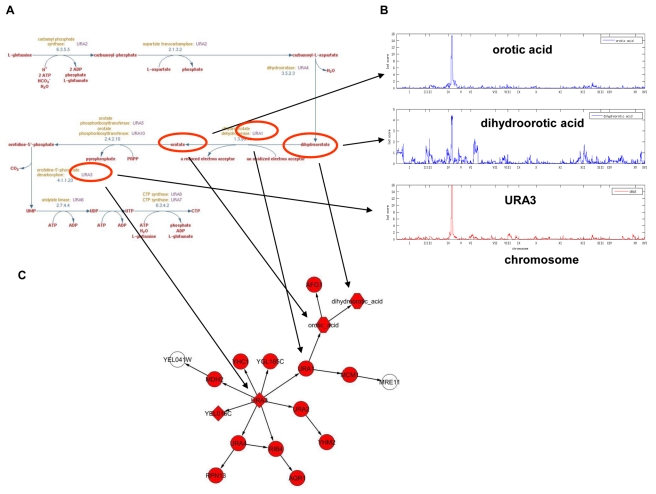
Relationship between metabolites and genes linked to eQTL hot spot 2 on Chromosome V. (A) De novo biosynthesis of pyrimidine pathway; (B) orotic acid and dihydroorotic acid concentrations are linked to the *URA3* locus; (C) *URA3* is predicted as the causal regulator for genes and metabolites linked to the eQTL hot spot. Red nodes are genes or metabolites whose variations are linked the Chromosome V locus. The shapes of the nodes follow the convention described in Figure 3.

The expression traits linked to this eQTL hot spot include *URA3*, a gene that is physically located in this hot spot region. From the BN, *URA3* is predicted as a causal regulator of this eQTL hot spot. A deletion of *URA3* was engineered in the parental strain RM11-1a as a selectable marker, and segregation of this locus among the BXR progeny is likely causal for expression variation of uracil biosynthesis genes linked to this eQTL hot spot [12]. Variation of two metabolites linked to this locus: dihydroorotic acid, which is converted to orotic acid by the enzyme Ura1p, and orotic acid itself, reflects the functional consequence of transcriptional variation in genes involved in de novo pyrimidine base biosynthetic processes on metabolite levels. The causal relationships between *URA1*, orotic acid, and dihydroorotic acid as well as the subnetwork for this eQTL hot spot recapitulate the known pyrimidine base biosynthesis pathway (Figure 4). This subnetwork not only captures the coregulation of gene expression and metabolite abundance, but also elucidates the mechanism of how genetic variation in *URA3* affects orotic acid and dihydroorotic acid levels.

#### Subnetwork linked to eQTL hot spot 3

**Figure 5:**
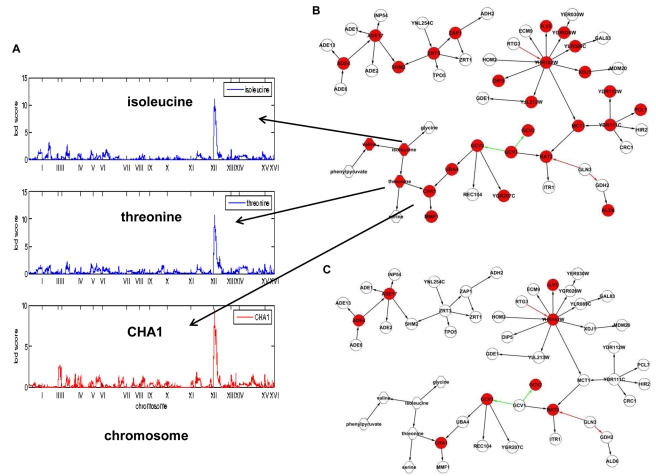
Genes and metabolites linked to eQTL hot spot 3 on Chromosome XIII. (A) Variations of the metabolites isoleucine and threonine are linked to this locus. (B) These two subnetworks comprise genes and metabolites enriched for linking to the Chromosome XIII locus. The larger network consists of both gene expression and metabolite nodes enriched for the GO biological process nitrogen compound metabolism. The smaller network is enriched for the GO biological process de novo IMP biosynthetic process. Red nodes are genes with eQTLs linked to the Chromosome 13 locus. (C) Expression levels of eight genes (in red) are different between *VPS9* knockout and the wild-type strains. The shapes of the nodes follow the convention described in Figure 3.

**Figure 6:**
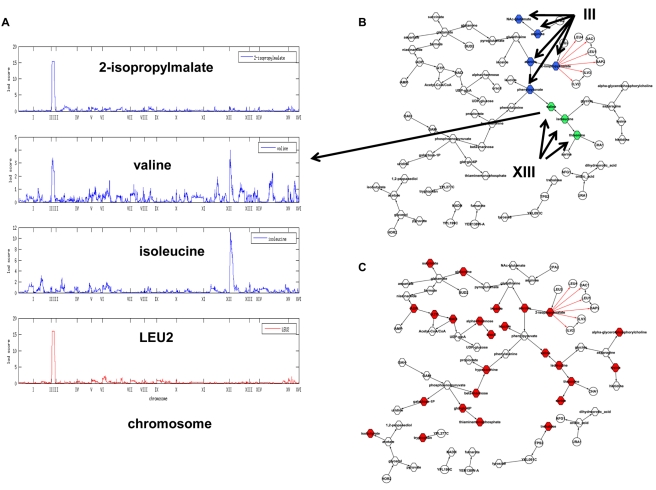
Metabolite subnetwork. (A) Variations in valine concentrations are linked to two eQTL hot spots; Chromosome III:100,000 and Chromosome XIII:70,000. (B) Most metabolites are connected. Valine connects to metabolites linked to eQTL hot spots at Chromosome III:100,000 (nodes in blue) and Chromosome XIII:70,000 (nodes in green). (C) 25 metabolites (in red) whose concentrations are different between *VPS9* knockout and the wild-type strains are in this subnetwork. This structure suggests that *VPS9* is causal for the variations of these metabolites. The shapes of the nodes follow the convention described in Figure 3.

Variations in the levels of isoleucine, threonine, and valine were linked to eQTL hot spot 3, along with the subnetwork in which these three metabolites reside. Little is known about the biological processes associated with this locus [13],[24]. However, we noted surprisingly that while the expression levels of six of seven genes in the isoleucine biosynthesis pathway were linked to eQTL hot spot 1 on Chromosome III, the concentrations of isoleucine and threonine linked to eQTL hot spot 3 (Table 1 and Figure 5a). We had previously shown that genes whose transcript levels linked to eQTL hot spot 1 on Chromosome III were enriched for amino acid biosynthesis pathways [13], with five amino acid and intermediate metabolites also linked to this locus (Table 1). These linked biomolecules included several regulators of branched-chain amino acid biosynthesis and the amino acids themselves. Interestingly, valine concentrations linked both to eQTL hot spots 1 and 3 (Figure 6A) along with valine associated metabolites (Figure 6B), suggesting that both loci may ultimately prove to be key regulators for a majority of amino acid levels in the BXR cross.

Two subnetworks were associated with eQTL hot spot 3 (Figure 5B). In the larger subnetwork, the metabolites isoleucine, valine, and threonine were inferred to connect through threonine to the expression levels of *CHA1* (Figure 5B), consistent with the known function of Cha1p as a catabolic serine/threonine deaminase, which is transcriptionally regulated by serine and threonine [40]. Expression levels of other amino acid catabolism genes (*BAT2*, *ILV5*, and *GCV1-3*) were also placed in this subnetwork, and the set of genes comprising this subnetwork was enriched for genes in the gene ontology (GO) Biological Process category “nitrogen compound metabolism” (Fisher exact test *p* = 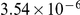). By contrast, the smaller subnetwork was enriched for genes in the GO Biological Process de novo inosine monophosphate (IMP) biosynthetic process category (Fisher exact test *p* = 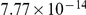
_)_. The known relationship between amino acid and purine nucleotide biosynthesis [41],[42] suggests a model in which a master regulator at eQTL hot spot 3 controls expression of both subnetworks of genes and metabolites.

Given that our network approach did not predict a causal regulator for eQTL hot spot 3, we examined whether *cis*-regulatory sequence variations in the BXR cross affected the expression of a gene located in this region and then whether such a gene was supported as causal for downstream targets also linked to this locus. *TAF13* was the only gene located in the eQTL hot spot 3 locus with *cis*-regulatory expression variation, but this gene was not connected to any of the inferred subnetworks associated with this hot spot.

Reasoning that *TAF13* was unlikely to be the causal regulator of the eQTL hot spot, we hypothesized instead that the underlying causal variant might lead directly to a protein activity change rather than to a change in transcript levels. To identify such protein-coding variants, we compared the genomes of BY and RM at this locus and found nonsynonymous changes in *YML096W*, *VPS9*, *ARG81*, *TSL1*, *CAC2*, and *NUP188*. We considered each of these genes as a candidate regulator for the eQTL hot spot 3 locus. To evaluate these candidates, we anticipated that for any true causal gene at the locus, the protein product of the gene would be necessary for maintaining wild-type metabolite levels in a single tester strain. As such, we experimentally tested knockout strains for each candidate gene in the BY background, comparing in each case the concentrations of metabolites with those of the wild type. The results, listed in Table S4 and Figure S5, revealed dramatic changes in metabolite levels for the knockout of the vacuolar transport regulator *VPS9*, compared to the other candidate genes, where the corresponding knockouts had modest to insignificant metabolite changes. Loss of *VPS9* was associated with changes in threonine, isoleucine, valine, and serine concentrations, something we would expect if *VPS9* was the causal regulator for this linkage hot spot, given amino acids linked to this hot spot reside in the corresponding subnetwork (Figure 6C). The *VPS9* deletion also affected ADP and ATP concentrations, consistent with the de novo IMP and purine nucleotide biosynthetic process associated with this locus, as discussed above. Many metabolites are interconnected in the network (Figure 6B) so that *VPS9* deletion has a broad effect on metabolite concentrations (Figure 6C).

We further profiled the effects the *VPS9* deletion had on the expression levels of the 16 genes in the small eQTL hot spot 3 subnetwork. We observed significant expression changes in the knockout relative to wild-type in eight of the 16 genes tested (*p*<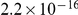) (Figure 5C; Tables S5 and S6), including those genes annotated in amino acid catabolism and nucleotide biosynthesis. Taken together, our results implicate *VPS9* as a major determinant of amino acid levels and expression of amino acid catabolism genes, with strong experimental support for sequence variation in *VPS9* serving as the causal factor underlying the changes in these biomolecules in the BXR cross.

#### Subnetwork linked to eQTL hot spot 4

**Figure 7:**
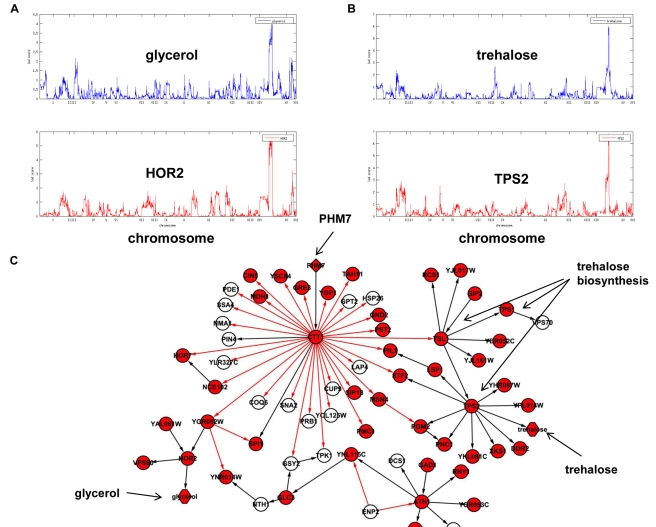
Genes and metabolites linked to eQTL hot spot 4 on Chromosome XV. (A) Variations in the metabolites glycerol and (B) trehalose are linked to this eQTL hot spot. (C) The part of the subnetwork associated with this eQTL hot spot consists of the causal regulator *PHM7* at the top, key TFs MSN4 and MSN2 (represented by *CTT1*), and the genes that encode for the trehalose synthesase complex. Red nodes are genes or metabolites with QTL linked to the Chromosome XV locus.

eQTL hot spot 4 has been identified by us and others as a major driver of expression differences in the BXR cross for genes involved in stress response [13],[24]. Previous work has investigated the role of sequence variation in *IRA2* [23] and *PHM7* [13] as causal regulators at this locus. Interestingly, though the levels of hundreds of transcripts coinherited with sequence variants at the Chromosome XV eQTL hot spot locus, the levels of proteins encoded by such transcripts did not generally show linkage to the locus [34], leading to speculation that the mRNA variation may not have appreciable downstream consequences. In our metabolite data, abundances of trehalose and glycerol, both implicated in the yeast stress response [43], were significantly linked to this locus (Figure 7A and 7B). Our network predicted *HOR2* expression as a determinant of glycerol levels, consistent with the known function of Hor2p in glycerol synthesis and its regulation by the stress response TF complex Msn2/4. In our network the metabolite trehalose was located in a subnetwork with *TPS2*, *TPS1*, and *TSL1* (Figure 7C), consistent with the known function of these genes as trehalose synthase components. *MSN2* was predicted by the network as an upstream regulator of trehalose synthesis (where *MSN2* activity was represented by *CTT1* in the network), recapitulating the known stress response function of Msn2p. Further upstream of this process, our network predicted *PHM7* as the major causal regulator of the entire subnetwork. Little is known about the function of Phm7p, but in support of a causal role for variation at this gene in control of stress response, we previously showed that a knockout of *PHM7* affects expression of many genes with linkage to the Chromosome XV eQTL hot spot 4 locus [13].

To validate our prediction that *PHM7* affects the abundance of stress response metabolites such as trehalose and glycerol in addition to stress response genes linked to the eQTL hot spot, we profiled metabolite levels in the *PHM7* knockout and wild-type strains (Methods). The abundance of trehalose in the *PHM7* knockout strain was 2.4× higher compared to the wild-type strain (*p* = 0.03), which was the largest fold change among all metabolites. However, the abundance of glycerol in the *PHM7* knockout strain did not significantly change. *PHM7* has a stronger effect on trehalose abundance than on glycerol abundance, which is consistent with the metQTL results that the metQTL LOD score of trehalose at the eQTL hot spot 4 locus is 6.03, while the metQTL LOD score of glycerol is 4.38.

**Figure 8:**
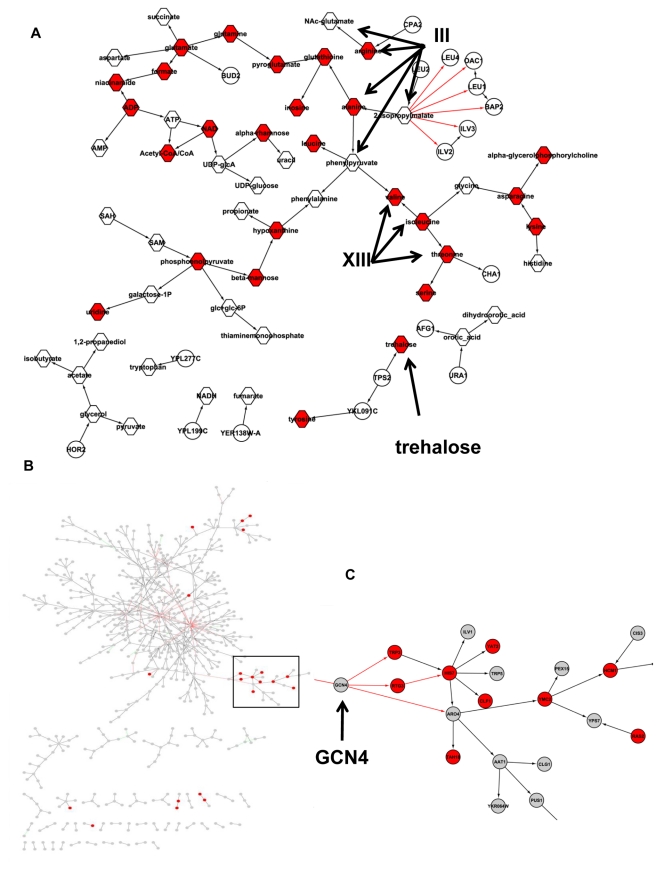
The *PHM7* knockout metabolite signature suggests interconnectivity of multiple eQTL hot spots. (A) The metabolite subnetwork is the same as the subnetwork depicted in Figure 6A. 27 metabolites (in red) whose concentrations differ between the *PHM7* knockout and the wild-type strains are in this subnetwork. In addition to trehalose, which is linked to the eQTL hot spot 4, the *PHM7* knockout metabolite signature includes metabolites whose concentrations are linked to eQTL hot spots 1 and 3 (on Chromosomes III and XIII, respectively), suggesting interactions among eQTL hot spots 1, 3 and 4, as we have previously predicted [44]. (B) The subnetworks for eQTL hot spot 4 (extracted using genes linked to eQTL hot spot 4) suggests that part of this network is regulated by both eQTL hot spots 2 and 4. Red nodes are genes whose expression values are linked to eQTL hot spot 2. (C) A zoomed-in view of the part of the network regulated by eQTL hot spots 2 and 4. The gene that links this part of the network to the rest of the subnetwork associated with eQTL hot spot 4 is *GCN4*, a master TF regulating amino acid biosynthesis.

In addition to trehalose, there were a total of 27 (out of 56) metabolites whose abundance levels were significantly different (*p*<0.05) between the knockout and wild-type strains (Table S7), including phosphoenolpyruvate (a key intermediate metabolite in glucolysis and gluconeogenesis) and a number of amino acids and their intermediates. These metabolites are closely associated with the metabolites whose abundance levels are linked to eQTL hot spots 1 and 3 (Figure 8A). Using a Bayesian partition method, we previously predicted a module of 83 genes including ILV6 is modulated by eQTL hot spots 1, 3, and 4 on Chromosomes III, XIII, and XV, respectively, with eQTL hot spot 1 also enriched for genes involved in amino acid metabolism [44]. Genes whose expression levels are linked to both eQTL hot spots 1 and 4, including HIS7, YMC2, and HCM1, are colocalized to the same subnetwork associated with eQTL hot spot 4 (Figure 8B). HIS7, an enzyme involved in histidine, purine, and pyramidine biosynthesis, is linked to the rest of the subnetwork through the amino acid biosynthesis regulator *GCN4* (Figure 8C). That the *PHM7* knockout metabolite signature contains amino acids and their intermediate metabolites linked to eQTL hot spots 1, 3, and 4 (Figure 8A), not only confirms the biological consequence of transcriptional changes and validates our prediction of the biological function of the eQTL hot spot 4 subnetwork, but it also validates our predictions of interconnectivity of eQTL hot spots 1, 3 and 4 [8].

Given the known *cis*-acting regulatory changes between BY and RM at the *PHM7* gene [45], together with the gene expression and metabolite profiles of the PHM7 knockout and wild-type strains, one interpretation of our identification of *PHM7* as the causal regulator of this stress response network is that its expression variation creates a stress condition that activates Msn2/4, which in turn activates stress response genes. Our network suggests regulatory relationships among stress response genes and metabolites, and enables emergent hypotheses about novel genes in the stress response pathway.

## Discussion

By integrating six different fundamental types of data, including RNA expression, DNA variation, DNA–protein binding, protein–metabolite interaction, and protein–protein interaction data, with metabolite data, we constructed a BN using an approach that simultaneously considers all of these data, with the resulting network providing a number of novel insights into the mechanisms of the eQTL hot spots in a segregating yeast population (the BXR cross). Importantly, we validated the biological consequences of the transcriptional variation linked to each of the four eQTL hot spots identified in the BXR cross to which metabolite levels were also linked. Our results indicate that the incorporation of metabolite levels into the network reconstruction process significantly enhanced the utility of the network-based models [46],[47]. While the integration of metabolite abundance and gene expression traits in a genetic context have been attempted in plants [48] and mouse [49], the main distinguishing characteristic of our study is the de novo construction of a global molecular network that simultaneously incorporates many different types of information (DNA, RNA, protein, and metabolite), along with known biochemical pathways as prior information. To aid in further understanding how we integrate these data to construct probabilistic causal networks, and to enhance the ability to repeat our results, we provide as Text S1 results of an in-depth description of the construction of the *URA3* subnetwork (Figure 4), using different types of data to assess the contributions of different data types to the predictive power of the network and to the identification of key modulators of important biological processes. We examined in detail all 4 eQTL hot spots that coincided with metQTLs. Our findings for eQTL hot spots 1 and 2 recapitulated well-known biological processes, and for eQTL hot spots 3 and 4 our predictions implicated novel genes as modulators of established biological processes, which we subsequently validated prospectively. Among the many predictions made by our network, we uncovered novel insights into the biological processes that in the BXR cross are responsible for variations in amino acid levels. While amino acid concentrations are known to be regulated by multiple processes (e.g., synthesis, degradation, recycle, and storage), our approach objectively identified that variations in concentrations of a number of amino acids in the BXR cross were affected by both the amino acid biosynthesis and degradation pathways. We predicted and prospectively validated *VPS9* as a major driver of amino acid concentrations via the amino acid degradation pathway. These results open novel and interesting questions about the mechanism by which sequence variation at this locus affects phenotype. *VPS9* is involved in vesicle-mediated vacuolar protein transport, and in *Saccharomyces cerevisiae*, the vacuole is the main compartment for amino acid storage, recycling, and cytosolic amino acid concentration maintenance [50]. The cellular effects of variation in *VPS9* are likely mediated by differential regulation of amino acid storage in the vacuole; we speculate that such storage changes may affect cytosolic amino acid pools that in turn have downstream consequences on transcript and protein levels of amino acid pathways, as has been shown for *CHA1* [40] and *GCV3* [51]. However, only with enhanced screening of all molecular states of the systems can we achieve a complete understanding of these processes. Thus, while the integrated BN elucidated some of the mechanistic underpinnings of the eQTL hot spots in the BXR cross, additional information will be required to more fully understand how processes perturbed in the BXR cross lead to phenotypic changes.

Despite lacking an exhaustive assessment of all molecular traits in the BXR cross, it is of particular note that the strong correlations we observed between gene expression and metabolite data may help resolve an ongoing debate regarding the functional consequences of gene expression regulation. While some reports indicate that gene expression levels and protein abundances are not well correlated [52], other reports indicate a high degree of correlation [53]. A recent proteomic study in the BXR cross demonstrated that a large number of protein levels are linked to eQTL hot spots [34], two of which (the eQTL hot spots 1 and 3) were highlighted in our present work. Metabolites are the final functional products of protein activity regulation. We showed that *PHM7* not only alters expression levels of stress response genes linked to eQTL hot spot 4, but also alters the abundance of trehalose, a metabolite product of the stress response genes. Our results demonstrate that gene expression and metabolite levels are not only strongly correlated, but that a significant proportion of that covariation can be explained by common genetic control. Given that variations in protein levels can result from sequence-specific transcriptional and translational regulation or from nonsequence-specific protein degradation, the integration of gene expression and metabolic traits can help dissect the complex processes that regulate protein levels.

The yeast growth conditions for metabolite profiling were the same as previously used to generate the gene expression data in the BXR cross [12]. Both gene expression and metabolite abundances are under strong genetic regulation and are linked to common eQTL hot spots (Table 1). When metabolite data were integrated with gene expression data, our resulting integrated network was able to recapitulate the mechanism of multiple known biological processes that in turn explained the connection between genes linked to the *LEU2* locus and genes with Leu3 binding sites, with the metabolite 2-isopropylmalate objectively identified as the key intermediate. These results also confirmed that changes in expression of stress response genes lead to changes in stress response metabolites such as trehalose. Therefore, the integration of the gene expression and metabolite data has provided new insight into common biological processes that are perturbed by genetic variation segregating in the BXR cross.

Going forward, as more technologies emerge that can generate large-scale data in different dimensions for low cost, we will achieve a more complete understanding of biological systems only if we integrate all of the information together to consider all of the different cellular components and how they interact with one another at the population level. For example, comprehensive proteomic data and protein phosphorylation data are needed and should be further integrated with other high throughput genomic and genetic data. For metabolites, their cellular abundances are not only affected by specific enzymes in related biochemical reactions, but they are also affected by proteins that bind them or transport them into different cellular compartments. Further research on how to integrate these data into networks is needed. In addition, there is an abundance of existing knowledge, such as genetic interactions and regulatory cascades, which can be converted into prior information and integrated with other data and priors. Further efforts in developing methods to integrate these diverse data and information are warranted. In more complex systems, we will need to consider the fundamental building blocks of a cell in the context of cell–cell interactions that lead to tissue-based networks, the interactions of tissues that lead to organ-based networks, and the interactions of organs in a given system to understand the physiological states of that system associated with complex phenotypes of interest, given these phenotypes emerge from this complex web of interacting networks [54]. Only by taking the full complement of raw data available on living systems can we move from the accumulation of knowledge to actual understanding, and from understanding, wisdom.

## Methods

### Strains in the Yeast BXR Cross and Growth Conditions

Yeast parental strains BY4716 (*MAT* **α** *lys2*Δ0) and RM11-1a (*MAT* **a** *leu2*Δ0 *ura3*Δ0 *HO:kan*) and 111 segregants of BXR cross [11] were provided by R. Brem. Auxotrophies, mating type, and G418 resistance were confirmed for all strains to be as previously reported [12]. Cells were grown under identical conditions as previously described [12]. Strains were freshly started from freezer stocks and stored at room temperature on synthetic complete medium plates for no longer than 1 wk before each experimental run. For each run, cells from the plates were precultured in 10 ml of synthetic complete media (Table S8) at 30°C with shaking for 24 h. These cultures were then diluted into 25 ml fresh synthetic complete media to an optical density of 0.005 to 0.02. This starting density was determined from previous growth rate measurements and empirical observations such that after overnight growth at 30°C, the cultures would be exponentially growing, i.e., at a cell density of less than 2×10^7^ cells/ml. Overnight cultures were diluted into 52 ml fresh synthetic complete medium to an optical density of 0.1, and incubated with shaking for approximately 5 h at 30°C. Starting at 3 h after dilution, optical density was monitored every 60 min. Cell suspensions were counted in a hemocytometer to obtain cell count per OD values and an estimate of cell-doubling time. Since some of the yeast strains produced flocculent cultures under these growth conditions, all cultures were diluted 5× into 0.25 ml PBS and sonicated three times on ice for 45 s using a Misonix sonicator 3000 equipped with a microprobe before optical density was determined and/or cells were counted. At an optical density of approximately 1.0, each exponentially growing culture was concentrated 10-fold by rapid centrifugation at room temperature and suspension of the cells in 5 ml of synthetic complete medium prewarmed to 30°C. These concentrated cell suspensions were then incubated at 30°C with shaking for 1 h. Metabolites were then immediately extracted from the cells in these concentrated suspensions.

### Yeast Knockout Strains and Growth Conditions

Yeast parental strain BY4742 (*MAT* **α** *his3*Δ1 *leu2*Δ0 *met15*Δ0 *ura3*Δ0) and six deletion strains derived from it (Δ*tsl1::kanMX*, Δ*nup188::kanMX*, Δ*cac2::kanMX*, Δ*yml096w::kanMX*, Δ*vps9::kanMX*, and Δ*arg81::kanMX*) were provided by Elton Young's lab, Department of Biochemistry, University of Washington, from a copy of the Yeast Deletion Consortium knockout collection prepared in Stanley Fields' lab, Department of Genome Sciences, University of Washington. Cells were grown under identical conditions as the BXR cross strains in synthetic complete medium, and metabolite extracts were also obtained and further processed in identical fashion (see below). Each experiment was repeated on three different days.

Yeast parental strain BY4743 (MATa/MATα his3Δ1/his3Δ1 leu2Δ0/leu2Δ0 lys2Δ0/+ met15Δ0/+ ura3Δ0/ura30) was obtained from ATCC (Manassas, Virginia), and the derived *PHM7* knockout strain 31775 (phm7::KanMX/phm7::KanMX) constructed by the Yeast Deletion Project [55] was obtained from Open Biosystems (Huntsville, Alabama). Cells were grown under identical condition as the *PHM7* knockout gene expression experiment [13], and metabolites were extracted as described below. Each experiment was repeated on three different days.

### Quantitative PCR

BY4742 and Δ*vps9* strains (both *MAT* **α**) were grown as described above and harvested by centrifugation in crushed ice when cells reached optical density of approximately 1.0. Total RNA was extracted using RNeasy mini-columns, transcribed with SMARTScribe Reverse Transcriptase (Clontech) from oligo(dT), and diluted 1,000×. Real-time PCR was run for 17 genes (including *VPS9*) associated with the Chromosome XIII eQTL hot spot subnetworks and *ACT1* internal standard gene (Table S6) on an ABI 7900HT instrument with 2× Sensimix dT (Quantance), primers at 0.2 µmol/l, and SYBR Green reagent. Relative expression was calculated using the ΔΔCt method with *ACT1* internal standards [56]. *TAF9* was used to estimate the false positive rate as 0.033.

### Metabolite Extraction

Intracellular metabolites were extracted using a modification of previously described methods [31],[57]. First, all intracellular metabolic processes were rapidly quenched by pipetting each concentrated cell suspension into 20 ml of rapidly mixing 60% (v/v) methanol at −40°C. Cells were rapidly (5 min) sedimented in a centrifuge precooled to −8°C and washed twice with 20 ml of the −40°C methanol. Metabolites were then extracted with boiling 75% (v/v) ethanol at 80°C and 0.25 ml dry volume of acid-washed glass beads (Sigma G1277), by vigorous vortexing for 30 s. The cell-glass bead slurry was incubated 3 min at 80°C, vortexed 30 s, and then placed on ice for 5 min. Large cellular debris and glass beads were removed by centrifugation at 2,000 g for 5 min. The resulting ethanolic extracts were clarified by three rounds of centrifugation at 14,000 g in a microcentrifuge. The clarified metabolite extracts were stored at −80°C until drying. Extracts were dried in a Savant Speed Vac under 150 mtorr vacuum in low retention microcentrifuge tubes. Dried metabolite extracts were stored at −80°C until preparation for NMR analysis.

### NMR Spectrum Acquisition and Metabolite Identification and Quantification

The process of NMR spectra acquisition and quantification follows the previously outlined procedure [29]. Dry metabolite extracts were dissolved in 0.7 ml deuterated 80 mM potassium phosphate buffer (containing 2 mM DSS-*d_6_* as an internal reference standard), and transferred to 100-mm 5-ml NMR tubes. NMR samples were stored in Varian 768AS auto-sampler at 8°C before and after NMR analyses. NMR data were acquired on the Varian 700 MHz NMR spectrometer at 25°C with one-dimensional proton pulse sequence. The water peak was suppressed by the WET pulse sequences [58]. For each sample, 512 acquisitions were acquired with 3 s of acquisition and 15 s of delay between pulses.

Analyses of NMR spectra were carried out using DataChord Spectrum Miner (One Moon Scientific, Inc.). Stacked NMR spectra were referenced to DSS-*d_6_* as 0 ppm, and peaks of each endogenous metabolite were checked against their reference spectra (about 700 common endogenous metabolites). Each metabolite usually displays multiple peaks, for example trehalose, shown in Figure S6. Overlapping peaks were quantified by peak area correction according to stoichiometric peak ratios for each metabolite.

### Genetic Variations as Anchors of Causal/Reactive Relationships in F2 Crosses

For three correlated variables 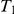, 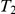, and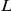, there are three groups of causal/reactive relationships among them as the following:
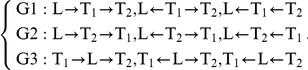
For example, the three graphs in the group G1, 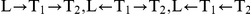, describe the same set of condition independent relationship that 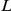 and 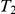 are independent conditioning on 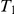. The three graphs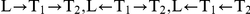 have the same probabilities and are called Markov equivalent. In an F2 cross, we can represent quantitative traits as 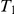 and 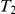, and the genetic locus as 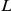. In an F2 cross experimental design, all F2 strains are under the same experimental condition. Therefore, the only source of variation in the quantitative traits 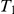 and 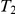 are genetic differences in 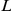, so that the relationships 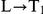 and 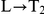 are plausible. On the other hand, the genetic variation in 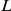 is stable and does not change during an F2 cross experiment, so that 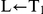 and 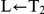 are not plausible. Thus in an F2 cross, only one graph in each of the three Markov equivalent groups above is plausible. We can simplify the above three groups as follows:
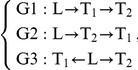
where the genetic locus 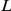 is the anchor in each causal/reactive relationship in a F2 cross.

### Inferring Causal Relationships between Quantitative Traits

For two quantitative traits 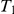 and 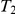 linked to the same locus 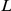 in the yeast cross, there are three basic relationships that are possible between the two traits relative to the DNA locus 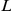 as described above. Either DNA variations at the locus 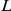 lead to changes in trait 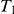 that in turn lead to changes in trait 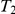, or variations at locus 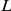 lead to changes in trait 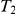 that in turn lead to changes in trait 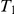, or variations at locus 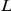 independently lead to changes in traits 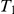 and 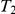, as previously described [14]. Assuming standard Markov properties for these basic relationships, the joint probability distributions corresponding to these three models, respectively, are:

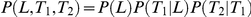

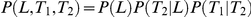

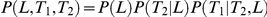
,

where the final term on the right-hand side of equation M3 reflects that the correlation between 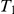 and 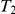 may be explained by other shared loci or common environmental influences, in addition to locus 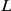. We assume Markov equivalence between 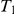 and 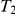 for model M3 so that 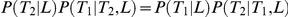. 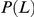 is the genotype probability distribution for locus 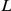 and is based on a previously described recombination model [59]. The random variables 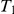 and 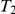 are taken to be normally distributed about each genotypic mean at the common locus 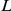, so that the likelihoods corresponding to each of the joint probability distributions are then based on the normal probability density function, with mean and variance for each component given by: (1) for 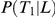 the mean and variance are 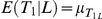 and 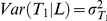, (2) for 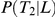 the mean and variance are 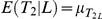 and 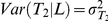, and (3) for 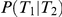 the mean and variance are 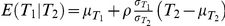 and 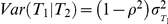, where 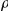 represents the correlation between 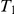 and 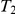, and 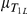 and 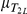 are the genotypic specific means for 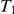 and 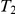, respectively. The mean and variance for 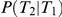 follow similarly from that given for 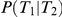. From these component pieces, the likelihoods for each model are formed by multiplying the densities for each of the component pieces across all of the individuals in the population [14]. The likelihoods are then compared among the different models in order to infer the most likely of the three. Because the number of model parameters among the models differs, a penalized function of the likelihood was used to avoid the bias against parsimony. The model with the smallest value of the penalized statistic
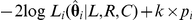
was chosen. Here, 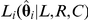 is the maximum likelihood for the *i*th model, *p_i_* is the number of parameters in the *i*th model, and *k* is a constant. In this instance we took the penalized statistic to be the Bayesian Information Criteria (BIC) where *k* is set to 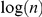, with *n* denoting the number of observations.

### Reconstructing Bayesian Network

BNs are directed acyclic graphs in which the edges of the graph are defined by conditional probabilities that characterize the distribution of states of each node given the state of its parents [60]. The network topology defines a partitioned joint probability distribution over all nodes in a network, such that the probability distribution of states of a node depends only on the states of its parent nodes: formally, a joint probability distribution 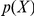 on a set of nodes 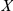 can be decomposed as 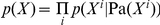, where 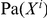 represents the parent set of 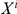. In our networks, each node represents a quantitative trait that can be a gene or a metabolite. These conditional probabilities reflect not only relationships between genes, but also the stochastic nature of these relationships, as well as noise in the data used to reconstruct the network.

Bayes formula allows us to determine the likelihood of a network model M given observed data D as a function of our prior belief that the model is correct and the probability of the observed data given the model: 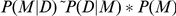. The number of possible network structures grows super-exponentially with the number of nodes, so an exhaustive search of all possible structures to find the one best supported by the data is not feasible, even for a relatively small number of nodes. We employed Monte Carlo Markov Chain (MCMC) [61] simulation to identify potentially thousands of different plausible networks, which are then combined to obtain a consensus network (see below). Each reconstruction begins with a null network. Small random changes are then made to the network by flipping, adding, or deleting individual edges, ultimately accepting those changes that lead to an overall improvement in the fit of the network to the data. We assess whether a change improves the network model using the BIC [62], which avoids overfitting by imposing a cost on the addition of new parameters. This is equivalent to imposing a lower prior probability 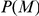 on models with larger numbers of parameters.

Even though edges in BNs are directed, we can't in general infer causal relationships from the structure directly. For example, in a network with two nodes, 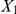 and 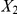, the two models 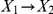 and 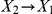 have equal probability distributions as 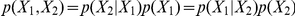. Thus, with correlation data itself, we can't infer whether 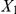 is causal for 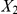 or vise versa. In the more general case, for a network with three nodes, 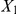, 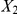, and 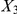, there are multiple groups of structures that are mathematically equivalent. For example, the following three different models, 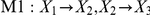, 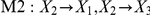, and 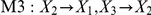, are Markov equivalent (which means that they all encode for the same conditional independent relationships). In the above case, all three structures encode the same conditional independent relationship, 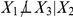, 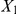 and 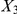 are independent conditioning on 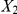, and they are mathematically equal
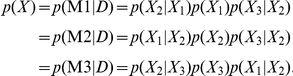
Thus, we can't infer whether 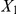 is causal for 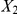 or visa-versa from these types of structures. However, there is a class of structures, V-shape structures (e.g., 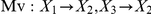), which have no Markov equivalent structure. In this case, we can infer causal relationships. There are more parameters to estimate in the Mv model than M1, M2, or M3, which means a large penalty in the BIC score for the Mv model. In practice, a large sample size is needed to differentiate the Mv model from the M1, M2, or M3 models.

#### Incorporating genetic data as a structure prior in the BN reconstruction process

In general, BNs can only be solved to Markov equivalent structures, so that it is often not possible to determine the causal direction of a link between two nodes even through BNs are directed graphs. However, the BN reconstruction algorithm can take advantage of the experimental cross design (or segregating populations more generally) by incorporating genetic data to break the symmetry among nodes in the network that lead to Markov equivalent structures, thereby providing a way to infer causal directions in the network in an unambiguous fashion [63]. We modified the reconstruction algorithm to incorporate genetic data as prior evidence that two quantitative traits may be causally related based on previously described causality test [63]. The genetic priors are constructed from three basic sources: (1) genes with cis-eQTLs [64] are allowed to be parent nodes of genes with coincident trans-eQTLs, 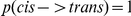, but genes with trans-eQTLs are not allowed to be parents of genes with cis-eQTLs, 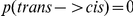. (2) The eQTL analysis described above was carried out to identify suggestive eQTLs for all expression traits [65] (LOD scores greater than 2.8, corresponding to less than 1 QTL expected by random across genome). Genes from this analysis with cis- or trans-eQTL were then tested individually for pleiotropic effects at each of their eQTL to determine whether any other genes in the set were driven by the same eQTL [59],[66]. If such pleiotropic effects were detected, the corresponding gene pair and locus giving rise to the pleiotropic effect were used to infer a causal/reactive or independent relationship based on a formal causality test [14]. The reliabilities of the inferred relationship between genes A and B at a given locus 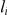, 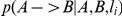, 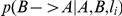, and 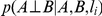, were then estimated by a standard bootstrapping procedure [67]. If an independent relationship is inferred (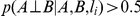), then the prior probability that gene A is a parent of gene B is scaled as 
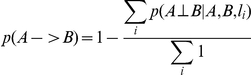
, where the sums are taken over all loci used to infer the relationship. If a causal or reactive relationship is inferred (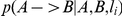 or 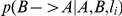 is greater than 0.5) then the prior probability is scaled as 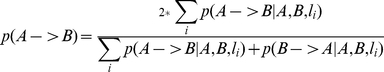. (3) If the causal/reactive relationship between genes A and B could not be determined by sources (1) or (2), then the complexity of the eQTL signature for each gene was taken into consideration. Genes with a simpler, albeit stronger eQTL signature (i.e., a small number of eQTLs that explains the genetic variance component for the gene, with a significant proportion of the overall variance explained by the genetic effects) were taken to be more likely to be causal compared to genes with more complex and possibly weaker eQTL signatures (i.e., a larger number of eQTLs explaining the genetic variance component for the gene, with less of the overall variance explained by the genetic effects). The structure prior that gene *A* is a parent of gene *B* was then taken to be 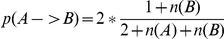, where *n*(*A*) and *n*(*B*) are the number of eQTLs with LOD scores greater than 2.8 for *A* and *B*, respectively. We have found that both information on cis-acting eQTLs (excluding edges into certain nodes) and information on trans-acting eQTLs (increasing the likelihood of some edges over others) improve the quality of the network reconstruction [68]. We note that in applying this particular version of the BN reconstruction algorithm (incorporating genetic information as a prior), if genetic information is not available or is ignored, the population is simply treated as a population with random genetic perturbations.

#### Incorporating TFBS and PPI data as network priors in the BN reconstruction process

Just as genetic data can be incorporated as a network prior in the BN reconstruction algorithm, we can similarly incorporate TFBS and protein complex data to establish prior evidence of a causal relationship between any gene pair. The PPI data were used to infer protein complexes to enhance the set of manually curated protein complex data [69]. Protein complexes were identified from the PPI data using the clique community analysis described above. The PPI-inferred protein complexes were then combined with the manually curated set, and each protein complex in this combined set was examined for common TF binding sites. If at least half of the genes in a protein complex carried a given TFBS, then all genes in the complex were included in the TFBS gene set as being under the control of the corresponding TF.

There are 119 TFs in the TFBS prediction set considered for this study [70], and 75 of these were included in the network because they met the criteria defined above for a gene to be included in the network (the others did not meet these criteria). Because TF activity can be regulated at the protein level (e.g., by phosphorylation), absence of detectable differential expression does not necessarily imply TF activity is not being actively regulated. Therefore, to account for the impact the 44 TFs that did not meet the criteria for inclusion in the network, may have on the expression of other genes, instead of introducing latent variables to represent activity of these TFs, we selected a gene from the set of genes predicted to respond to each of these TFs to represent the activity of the TF in the following way: (1) select the top five genes within the TF's responding gene set that were included in the network and that were supported as causal for the most genes in the set; and (2) select the gene with the highest LOD score at the common locus shared by the top five genes to represent the QTL signature of the TF. This same procedure was carried out for those protein complexes that were included in the TFBS set, as described above.

Given that the scale-free property is a general property of biological networks as shown in the main text and by others [71], we incorporated the enhanced TFBS set into the network reconstruction process by constructing scale-free priors, in a manner similar to the scale-free priors others have constructed to integrate expression and genetic data [72]. Given a TF 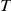, and a set of genes, 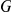, that contain the binding site of 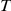, we define the TF prior, 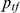, so that it is proportional to the number of expression traits correlated with the TF expression levels, for genes carrying the corresponding TFBS:
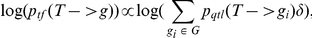
where 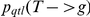 is the prior for the QTL and 
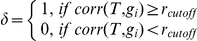
. The correlation cutoff 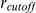 was determined by permuting the data and then selecting the maximum correlation values in the permuted datasets (corresponding to a 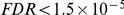). This form of the structure prior favors TFs that have a large number of correlated responding genes.

From this set of priors computed from the extended TFBS set, only non-negative priors were used to reconstruct the BN. This resulted in scale-free priors from 18 TFs and five protein complexes being incorporated in the network reconstruction process as previously described [13]. It is of note that the five protein complexes incorporated into the network reconstruction process were all large, with one representing the spliceosome and the other four representing the cytoplasmic and mitochondria ribosomes. For those protein complexes that could not be integrated into the network reconstruction process using scale-free priors, uniform priors were used for pairs of genes in these complexes (i.e., 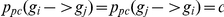).

#### Deriving structure priors from KEGG chemical reactions

81 XML files describing biochemical pathways in yeast were downloaded from ftp://ftp.genome.jp/pub/kegg/xml/organisms/sce/. 1,061 chemical reactions and associated catalyzing enzymes were parsed out, which were converted to 2,252 pairs of metabolite–enzyme relationships (shown in Figure S7). These relationships are stored in an adjacency matrix where a 1 in a cell represents a direct connection between the metabolite and the enzyme. The shortest distance 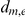 from an enzyme 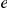 to a metabolite 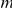 is calculated using the repeated matrix multiplication algorithm. The structure prior for the gene expression of an enzyme 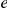 affecting a metabolite concentration is related to their shortest distance 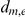 as 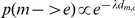. The shorter the distance, the stronger the prior.

#### Averaging network models

Searching optimal BN structures given a dataset is an NP-hard problem. We employed an MCMC method to do local search of optimal structures. As the method is stochastic, the resulting structure will be different for each run. In our process, 1,000 BNs were reconstructed using different random seeds to start the stochastic reconstruction process. From the resulting set of 1,000 networks generated by this process, edges that appeared in greater than 30% of the networks were used to define a consensus network. A 30% cutoff threshold for edge inclusion was based on our simulation study [68], where a 30% cutoff yields the best tradeoff between recall rate and precision. The consensus network resulting from the averaging process may not be a BN (a directed acyclic graph). To ensure the consensus network structure is a directed acyclic graph, edges in this consensus network were removed if and only if (1) the edge was involved in a loop, and (2) the edge was the most weakly supported of all edges making up the loop.

### Bayesian Network for the Yeast F2 Cross

The same 3,662 informative genes used previously [13] and 56 metabolites were included in the network reconstruction process using a BN reconstruction software program based on a previously described algorithm [63],[68] as outlined above. One thousand BNs were reconstructed using different random seeds to start the reconstruction process. From the resulting set of 1,000 networks generated by this process, edges that appeared in greater than 30% of the networks were used to define a consensus network. Our previous simulation study shows that the 30% inclusion threshold results in a stable structure and achieves the best tradeoff between precision and recall [68]. The histogram of percentage of occurrences of all potential edges shows that 30% is a reasonable cutoff threshold for inclusion (Figure S8). Edges in this consensus network were removed if (1) the edge was involved in a loop, and (2) the edge was the most weakly supported of all edges making up the loop. The genetic, TFBS, and PPI data were used to derive structure priors as previously described (details described above in Methods) [13]. Structure priors for metabolites and genes are derived from KEGG chemical reactions as described above.

All data and software used to construct the BNs described herein are available at http://www.mssm.edu/research/institutes/genomics-institute/rimbanet.

### Extracting a Subnetwork from Bayesian Network

Subnetworks for sets of genes were constructed as follows. Genes in the input set were used as seeds and the direct neighbors of seeds were identified. Seeds and their direct neighbors define the nodes of a given subnetwork. Links between nodes in the subnetworks are the same as in the complete BN.

## Supporting Information

Figure S1 Comparison of cis-eQTLs identified in the same yeast BXR cross under glucose and ethanol growth conditions.
(DOCX)

Figure S2 1D proton NMR spectra of BY and RM yeast strains.
(DOCX)

Figure S3 The distributions of gene expression variations among 111 segregants for: (a) *LEU2*; (b) *LEU3*.
(DOCX)

Figure S4 Relationship between arginine, NAc-glutamate, and genes linked to the eQTL hot spot on Chromosome III.
(DOCX)

Figure S5 Number of metabolites (*y*-axis) whose concentrations are significantly different (*p*<0.05) between knockout and wild-type strains.
(DOCX)

Figure S6 Illustration of identifying an endogenous metabolite in the RM strain sample (bottom), for example, based on a reference spectrum of trehalose (top).
(DOCX)

Figure S7 A global view of pairwise relationships of metabolites and catalyzing enzymes parsed from the KEGG biochemical pathway database.
(DOCX)

Figure S8 Histogram of percentages of occurrence of all potential edges.
(DOCX)

Figure S9 Trait values of nodes compared with genotype data for the URA3 subnetwork.
(DOCX)

Figure S10 BN reconstruction process using only trait data.
(DOCX)

Figure S11 BN reconstructed using only trait data.
(DOCX)

Figure S12 BN reconstruction process using trait data and priors derived from other types of data.
(DOCX)

Figure S13 BN reconstructed using trait data and priors derived from other types of data.
(DOCX)

Table S1 Comparison of genes linked to eQTL hot spots.
(DOCX)

Table S2 Averaged intracellular metabolite concentrations of all segregants.
(DOCX)

Table S3 Metabolites with significant concentration differences (Wilcoxon test *p*<0.005) between parental BY and RM strains.
(DOCX)

Table S4 Concentrations of 25 metabolites are different (*t*-test *p*<0.05) between *VPS9* knockout and wild-type strains.
(DOCX)

Table S5 Expression levels of eight genes are different (*t*-test *p*<0.01) between *VPS9* knockout and wild-type strains.
(DOCX)

Table S6 Primers used for real-time PCR quantification of Chromosome XIII hotspot subnetwork genes.
(DOCX)

Table S7 Abundances of 27 metabolites are different (*t*-test *p*<0.05) between *PHM7* knockout and wild-type strains.
(DOCX)

Table S8 Composition of the synthetic complete medium.
(DOCX)

Table S9 Correlation coefficients of all pairs of nodes in the URA3 subnetwork.
(DOCX)

Table S10 Mutual information for all pairs of nodes in the URA3 subnetwork.
(DOCX)

Table S11 Causality test results for the 18 nodes in the URA3 network.
(DOCX)

Text S1 An example in constructing BNs to make novel discoveries.
(DOC)

## Abbreviations

BN: Bayesian network
eQTL: expression quantitative trait loci
FDR: false discovery rate
IMP: inosine monophosphate
metQTL: metabolite quantitative trait loci
MS: mass spectrometry
NAc-glutamate: N-acetyl-glutamate
qNMR: quantitative nuclear magnetic resonance
TF: transcription factor
