# Supplementary material for: Stitching together Multiple Data Dimensions Reveals Interacting Metabolomic and Transcriptomic Networks That Modulate Cell Regulation
Source: PLoS Biol. 2012 Apr 3;10(4):e1001301. doi: 10.1371/journal.pbio.1001301 (PMC3317911; doi:10.1371/journal.pbio.1001301)
Supplement: Figure S1 — Comparison of cis-eQTLs identified in the same yeast BXR cross under glucose and ethanol growth conditions. (DOCX) [file pbio.1001301.s001.docx]

**
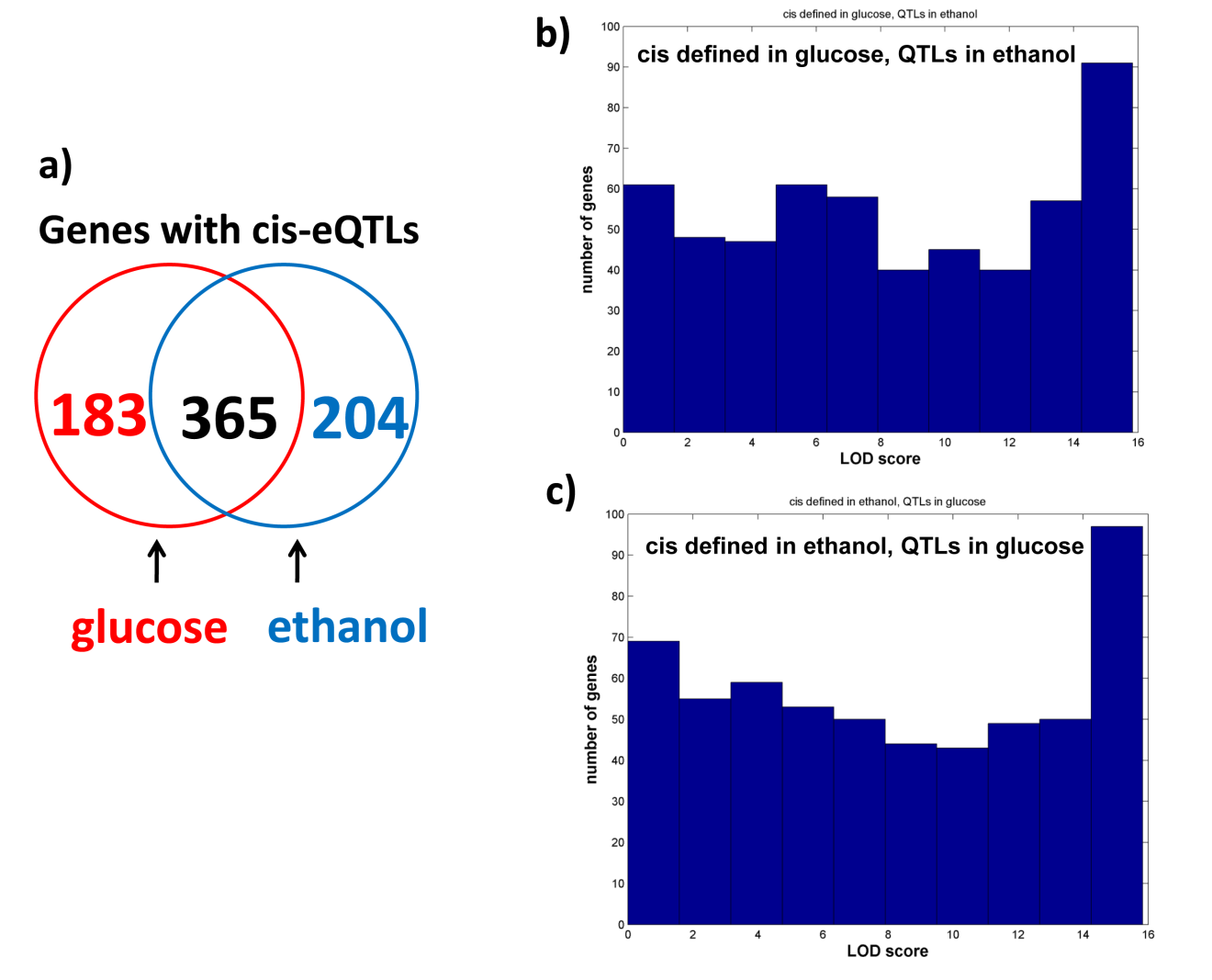
**

**C**

**B**

**A**

**Figure S1.** Comparison of cis eQTLs identified in the same yeast BXR cross under glucose and ethanol growth conditions. a) Half of the total cis eQTLs are common for both conditions, while the other half are unique to one growth condition. b) and c) are the distributions of eQTL LOD scores under one condition for genes with cis eQTLs detected in the other condition.
