# Supplementary material for: Stitching together Multiple Data Dimensions Reveals Interacting Metabolomic and Transcriptomic Networks That Modulate Cell Regulation
Source: PLoS Biol. 2012 Apr 3;10(4):e1001301. doi: 10.1371/journal.pbio.1001301 (PMC3317911; doi:10.1371/journal.pbio.1001301)
Supplement: Figure S2 — 1D proton NMR spectra of BY and RM yeast strains. (DOCX) [file pbio.1001301.s002.docx]

**
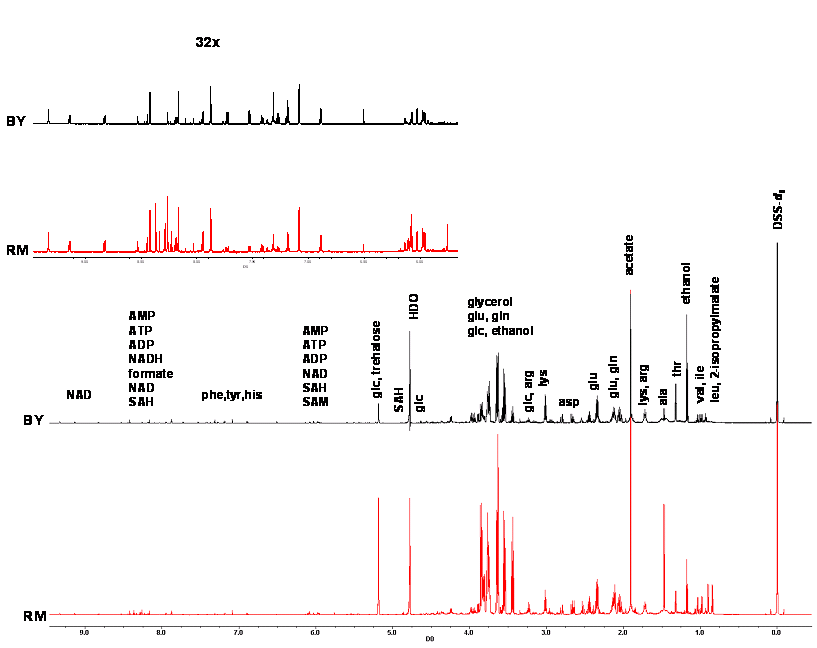
**

**Figure S2.** 1D proton NMR spectra of BY and RM yeast strains. The insert of the upper 2 traces are a 32× expansion of the down field region (i.e. left side of the bottom spectra).
