# Supplementary material for: Stitching together Multiple Data Dimensions Reveals Interacting Metabolomic and Transcriptomic Networks That Modulate Cell Regulation
Source: PLoS Biol. 2012 Apr 3;10(4):e1001301. doi: 10.1371/journal.pbio.1001301 (PMC3317911; doi:10.1371/journal.pbio.1001301)
Supplement: Figure S3 — The distributions of gene expression variations among 111 segregants for: (a) LEU2; (b) LEU3. (DOCX) [file pbio.1001301.s003.docx]

**
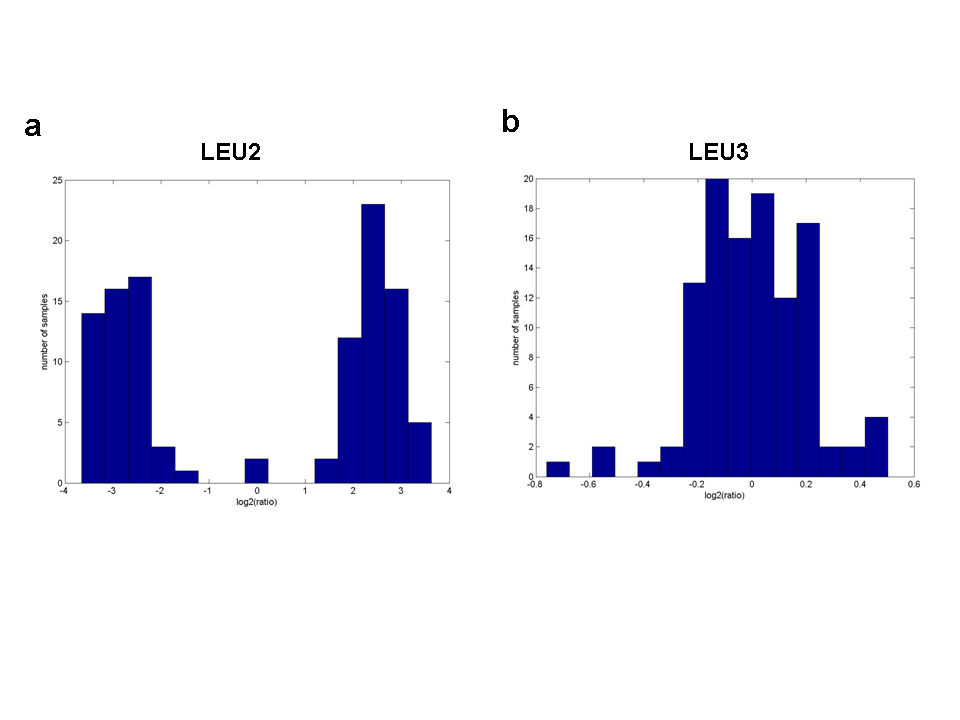
**

**B**

**A**

**Figure S3.**  The distributions of gene expression variations among 111 segregants for: a) *LEU2*; b) *LEU3*. *LEU2* expression varied significantly and correlated with genotypes at the *LEU2* locus. The variation of *LEU3* expression levels was small and was not linked to any locus.
