# Supplementary material for: Stitching together Multiple Data Dimensions Reveals Interacting Metabolomic and Transcriptomic Networks That Modulate Cell Regulation
Source: PLoS Biol. 2012 Apr 3;10(4):e1001301. doi: 10.1371/journal.pbio.1001301 (PMC3317911; doi:10.1371/journal.pbio.1001301)
Supplement: Figure S4 — Relationship between arginine, NAc-glutamate, and genes linked to the eQTL hot spot on Chromosome III. (DOCX) [file pbio.1001301.s004.docx]

**
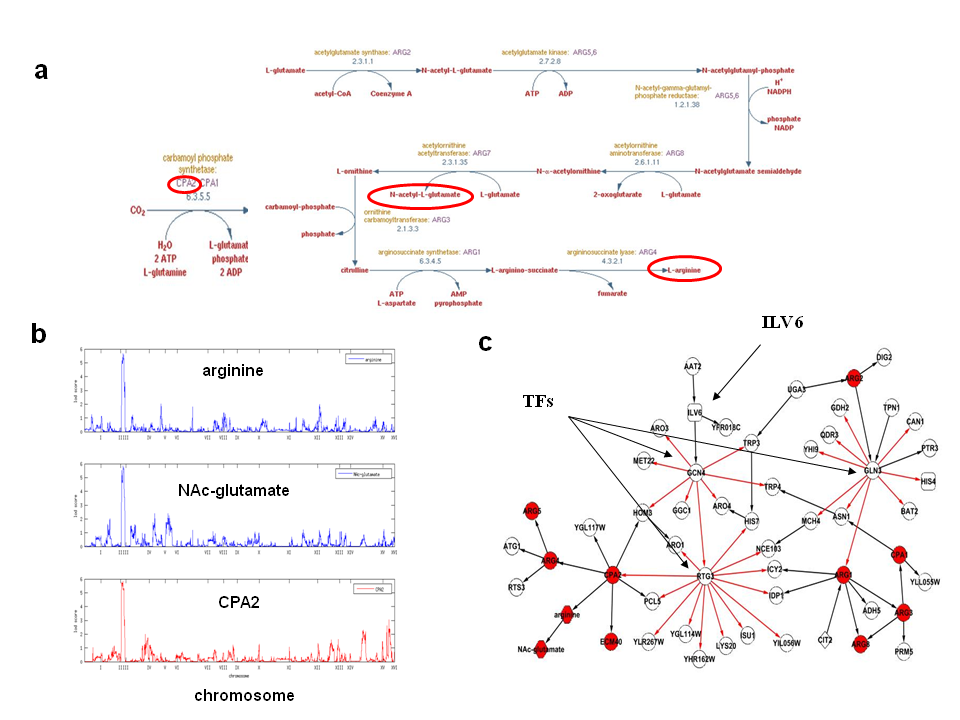
**

**C**

**B**

**A**

# Figure S4. Relationship between arginine, NAc-glutamate and genes linked to the eQTL hot spot on chromosome III. a) The Arginine biosynthesis pathway; b) QTL plots for metabolites and genes in the arginine biosynthesis pathway (red circles); c) Arginine, NAc-glutamate and genes involved in arginine biosynthesis pathway (red nodes) are under regulation of *ILV6* and multiple transcription factors *GCN4*, *RTG3* and *GLN3*.
