# Supplementary material for: Stitching together Multiple Data Dimensions Reveals Interacting Metabolomic and Transcriptomic Networks That Modulate Cell Regulation
Source: PLoS Biol. 2012 Apr 3;10(4):e1001301. doi: 10.1371/journal.pbio.1001301 (PMC3317911; doi:10.1371/journal.pbio.1001301)
Supplement: Figure S5 — Number of metabolites (y-axis) whose concentrations are significantly different (p<0.05) between knockout and wild-type strains. (DOCX) [file pbio.1001301.s005.docx]

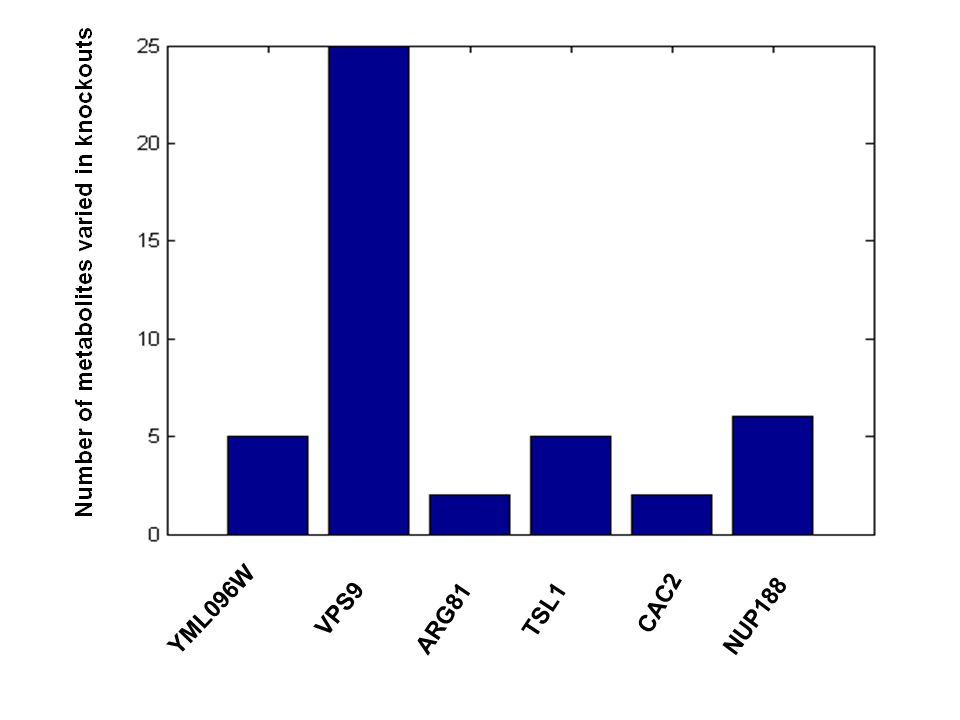


**Figure S5.** Number of metabolites (y-axis) whose concentrations are significantly different (p < 0.05) between knockout and wild type strains. The same 56 metabolites quantified in the BXR cross were tested. The number of metabolites with concentrations altered in the VPS9 knockout is much higher than in all other knockouts tested.
