# Supplementary material for: Stitching together Multiple Data Dimensions Reveals Interacting Metabolomic and Transcriptomic Networks That Modulate Cell Regulation
Source: PLoS Biol. 2012 Apr 3;10(4):e1001301. doi: 10.1371/journal.pbio.1001301 (PMC3317911; doi:10.1371/journal.pbio.1001301)
Supplement: Figure S6 — Illustration of identifying an endogenous metabolite in the RM strain sample (bottom), for example, based on a reference spectrum of trehalose (top). (DOCX) [file pbio.1001301.s006.docx]

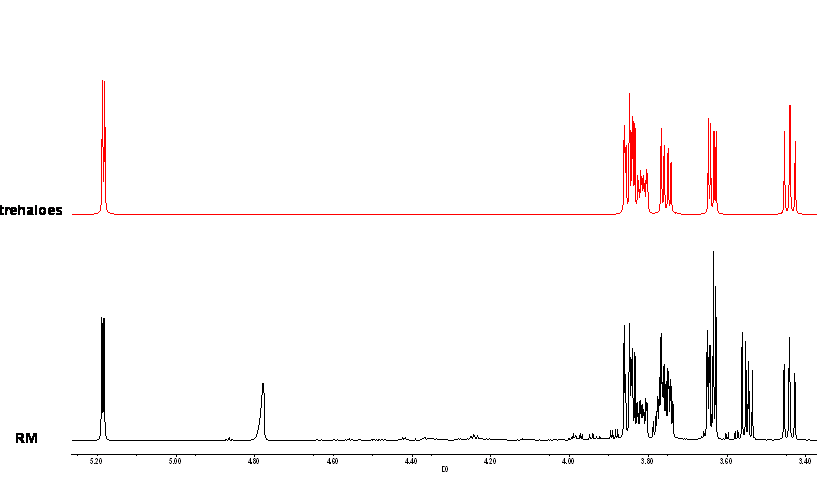


**Figure S6.** Illustration of identifying an endogenous metabolite in the RM strain sample (bottom), for example, based on a reference spectrum of trehalose (top).
