# Supplementary material for: Stitching together Multiple Data Dimensions Reveals Interacting Metabolomic and Transcriptomic Networks That Modulate Cell Regulation
Source: PLoS Biol. 2012 Apr 3;10(4):e1001301. doi: 10.1371/journal.pbio.1001301 (PMC3317911; doi:10.1371/journal.pbio.1001301)
Supplement: Figure S7 — A global view of pairwise relationships of metabolites and catalyzing enzymes parsed from the KEGG biochemical pathway database. (DOCX) [file pbio.1001301.s007.docx]

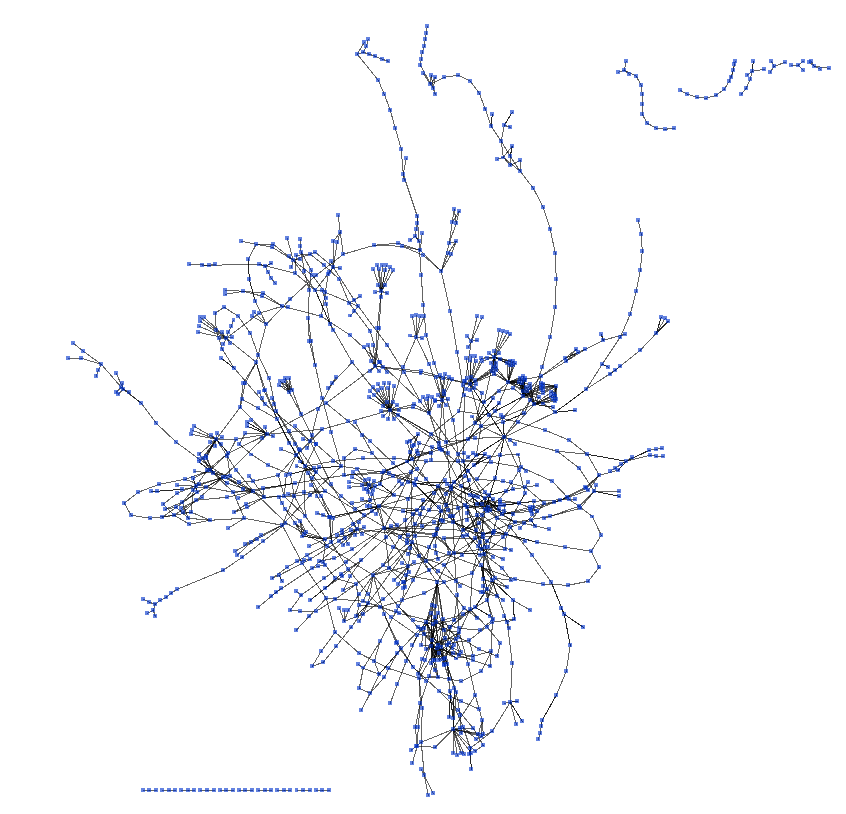


**Figure S7.** A global view of pairwise relationships of metabolites and catalyzing enzymes parsed from the KEGG biochemical pathway database.
