# Supplementary material for: Stitching together Multiple Data Dimensions Reveals Interacting Metabolomic and Transcriptomic Networks That Modulate Cell Regulation
Source: PLoS Biol. 2012 Apr 3;10(4):e1001301. doi: 10.1371/journal.pbio.1001301 (PMC3317911; doi:10.1371/journal.pbio.1001301)
Supplement: Figure S8 — Histogram of percentages of occurrence of all potential edges. (DOCX) [file pbio.1001301.s008.docx]

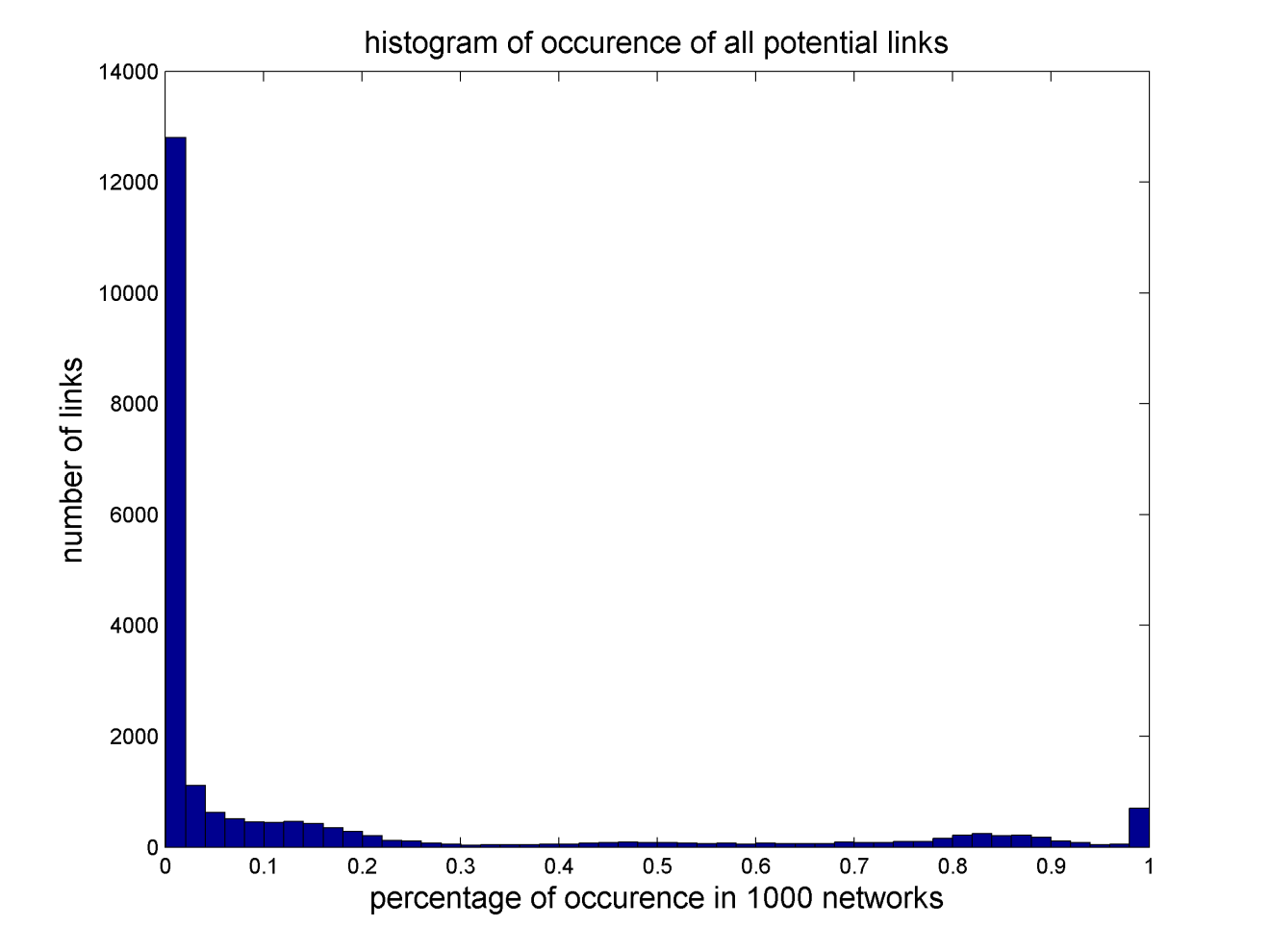


**Figure S8.** Histogram of percentages of occurrence of all potential edges. 30% is the natural threshold for edge inclusion.
