# Supplementary figures and images for: Stitching together Multiple Data Dimensions Reveals Interacting Metabolomic and Transcriptomic Networks That Modulate Cell Regulation
Source: PLoS Biol. 2012 Apr 3;10(4):e1001301. doi: 10.1371/journal.pbio.1001301 (PMC3317911; doi:10.1371/journal.pbio.1001301)

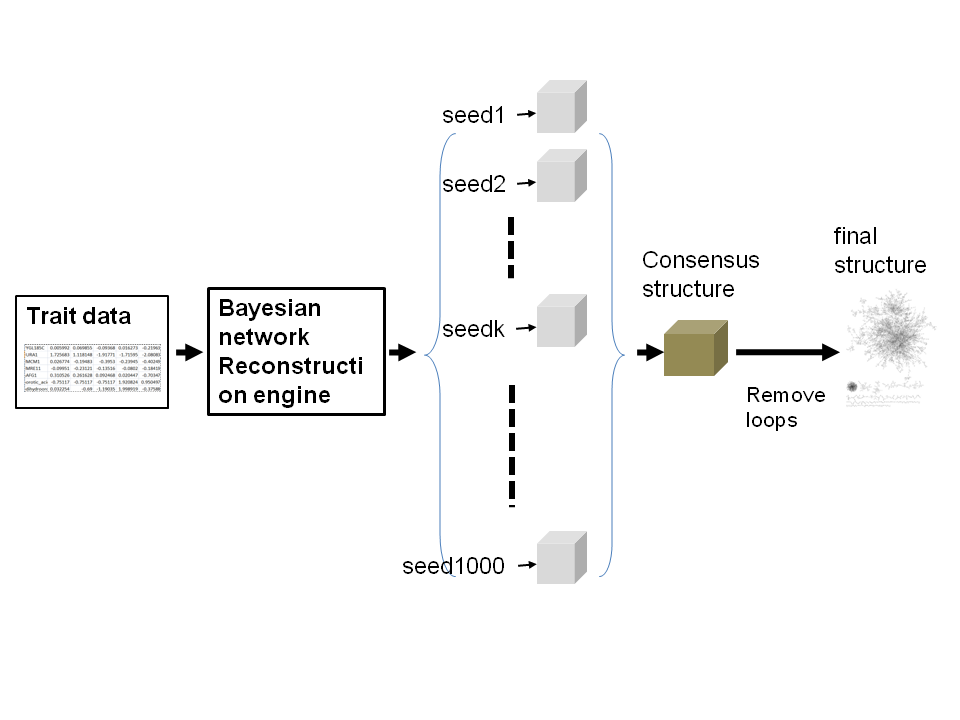


**Figure S10**. Bayesian network reconstruction process using only trait data.

Supplement: Figure S10 — BN reconstruction process using only trait data. (DOCX) [file pbio.1001301.s010.docx]

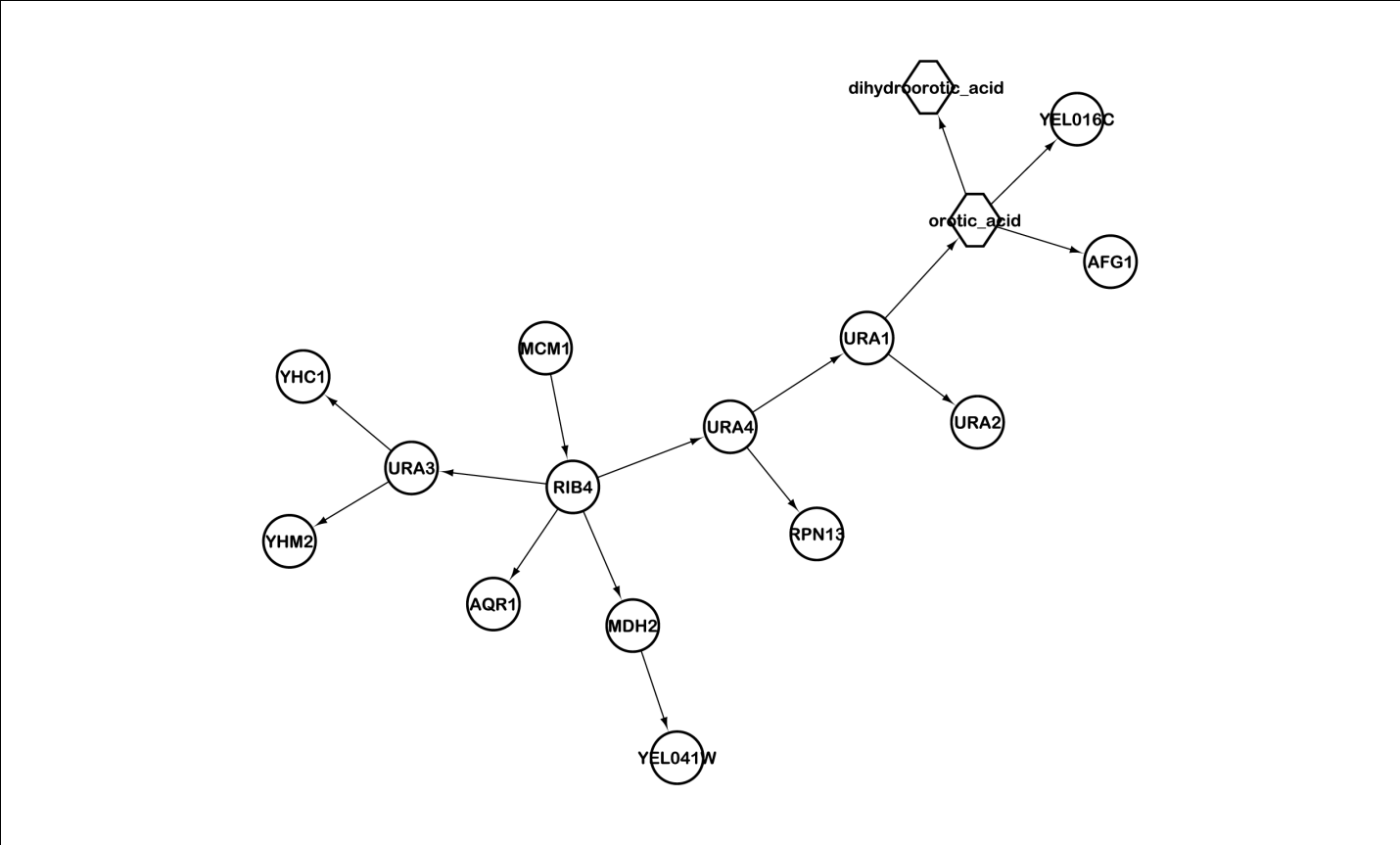


**Figure S11**. Bayesian network reconstructed using only trait data.

Supplement: Figure S11 — BN reconstructed using only trait data. (DOCX) [file pbio.1001301.s011.docx]
