# Supplementary material for: Stitching together Multiple Data Dimensions Reveals Interacting Metabolomic and Transcriptomic Networks That Modulate Cell Regulation
Source: PLoS Biol. 2012 Apr 3;10(4):e1001301. doi: 10.1371/journal.pbio.1001301 (PMC3317911; doi:10.1371/journal.pbio.1001301)
Supplement: Table S1 — Comparison of genes linked to eQTL hot spots. (DOCX) [file pbio.1001301.s014.docx]

**Table S1.** Comparison of genes linked to eQTL hot spots [5] based on two data sets for the same yeast BXR cross under similar growth conditions: Brem’s data [6] and Smith’s glucose data [7].

| **hot spot position (chromosome:position)** | **genes in Brem's data** | **genes in Smith's glucose data** | **overlap** | **p-value** |
| --- | --- | --- | --- | --- |
| II:390000 | 43 | 43 | 6 | 2.55E-06 |
| II:560000 | 400 | 130 | 42 | 1.88E-14 |
| II:710000 | 6 | 4 | 1 | 0.0053458 |
| III:100000 | 203 | 57 | 36 | 1.71E-35 |
| III:230000 | 11 | 11 | 4 | 6.43E-09 |
| V:110000 | 41 | 30 | 13 | 4.11E-20 |
| VIII:130000 | 31 | 25 | 11 | 9.82E-19 |
| XII:680000 | 154 | 158 | 59 | 2.71E-49 |
| XII:1070000 | 35 | 3 | 0 | 1 |
| XIII:70000 | 41 | 34 | 5 | 1.14E-05 |
| XIV:503000 | 278 | 313 | 165 | 5.89E-135 |
| XV:180000 | 343 | 1074 | 202 | 1.94E-47 |
| XV:590000 | 33 | 24 | 8 | 2.35E-12 |
