# Supplementary material for: Stitching together Multiple Data Dimensions Reveals Interacting Metabolomic and Transcriptomic Networks That Modulate Cell Regulation
Source: PLoS Biol. 2012 Apr 3;10(4):e1001301. doi: 10.1371/journal.pbio.1001301 (PMC3317911; doi:10.1371/journal.pbio.1001301)
Supplement: Table S2 — Averaged intracellular metabolite concentrations of all segregants. (DOCX) [file pbio.1001301.s015.docx]

**Table S2.** Averaged intracellular metabolite concentrations of all segregants. The derived intracellular concentration is based on cell volume estimation $2.9\times10^{-14}$L.

| **Metabolite** | **Measured concentration** | **Derived concentration** |
| --- | --- | --- |
|  | **(nmoles/cell)** | **(mM)** |
| orotic acid | 2.97E-09 | 1.02E-01 |
| dihydroorotic acid | 7.39E-09 | 2.55E-01 |
| UDP-glucose | 1.56E-08 | 5.36E-01 |
| phenylpyruvate | 1.01E-08 | 3.49E-01 |
| 1,2-propanediol | 2.14E-09 | 7.39E-02 |
| 2-isopropylmalate | 4.94E-08 | 1.70E+00 |
| acetate | 3.29E-07 | 1.14E+01 |
| Acetyl-CoA/CoA | 2.08E-09 | 7.18E-02 |
| ADP | 6.25E-09 | 2.15E-01 |
| alanine | 2.00E-07 | 6.90E+00 |
| alpha-glycerolphosphorylcholine | 4.95E-09 | 1.71E-01 |
| alpha-rhamnose | 3.49E-09 | 1.20E-01 |
| AMP | 1.40E-09 | 4.81E-02 |
| arginine | 1.32E-07 | 4.54E+00 |
| asparagine | 3.39E-08 | 1.17E+00 |
| aspartate | 2.98E-07 | 1.03E+01 |
| ATP | 1.88E-08 | 6.49E-01 |
| beta-mannose | 1.78E-08 | 6.15E-01 |
| formate | 7.53E-09 | 2.60E-01 |
| fumarate | 3.12E-09 | 1.08E-01 |
| galactose-1P | 4.44E-08 | 1.53E+00 |
| glc+glc-6P | 1.06E-07 | 3.65E+00 |
| glutamate | 9.55E-07 | 3.29E+01 |
| glutamine | 3.11E-07 | 1.07E+01 |
| glutathioine | 9.36E-08 | 3.23E+00 |
| glycerol | 6.59E-07 | 2.27E+01 |
| glycine | 1.37E-07 | 4.72E+00 |
| histidine | 8.35E-08 | 2.88E+00 |
| hypoxanthine | 3.23E-09 | 1.11E-01 |
| inosine | 4.75E-09 | 1.64E-01 |
| isobutyrate | 2.94E-09 | 1.01E-01 |
| isoleucine | 4.36E-08 | 1.50E+00 |
| leucine | 6.63E-09 | 2.29E-01 |
| lysine | 4.36E-07 | 1.50E+01 |
| NAc-glutamate | 1.57E-08 | 5.42E-01 |
| NAD | 4.11E-08 | 1.42E+00 |
| NADH | 3.47E-09 | 1.20E-01 |
| niacinamide | 1.68E-09 | 5.78E-02 |
| phenylalanine | 1.12E-08 | 3.86E-01 |
| phosphoenolpyruvate | 7.64E-09 | 2.63E-01 |
| propionate | 7.21E-09 | 2.49E-01 |
| pyroglutamate | 1.55E-08 | 5.35E-01 |
| pyruvate | 8.72E-09 | 3.01E-01 |
| SAH | 4.18E-08 | 1.44E+00 |
| SAM | 1.90E-08 | 6.56E-01 |
| serine | 2.51E-07 | 8.64E+00 |
| succinate | 1.25E-08 | 4.31E-01 |
| thiaminemonophosphate | 2.04E-09 | 7.02E-02 |
| threonine | 2.04E-07 | 7.04E+00 |
| trehalose | 3.17E-07 | 1.09E+01 |
| tryptophan | 2.01E-08 | 6.94E-01 |
| tyrosine | 1.69E-08 | 5.84E-01 |
| UDP-glcA | 1.43E-08 | 4.95E-01 |
| uracil | 2.31E-09 | 7.96E-02 |
| uridine | 1.88E-09 | 6.48E-02 |
| valine | 8.46E-08 | 2.92E+00 |
|  |  |  |
| **mean ratios**: |  |  |
| energy charge | 0.833096 |  |
| NAD/NADH | 20.022 |  |
