# Supplementary material for: Stitching together Multiple Data Dimensions Reveals Interacting Metabolomic and Transcriptomic Networks That Modulate Cell Regulation
Source: PLoS Biol. 2012 Apr 3;10(4):e1001301. doi: 10.1371/journal.pbio.1001301 (PMC3317911; doi:10.1371/journal.pbio.1001301)
Supplement: Table S3 — Metabolites with significant concentration differences (Wilcoxon test p<0.005) between parental BY and RM strains. (DOCX) [file pbio.1001301.s016.docx]

**Table S3.** Metabolites with significant concentration differences (Wilcoxon test p-value <0.005) between parental BY and RM strains.

| **Metabolite** | **p-value** |
| --- | --- |
| orotic acid | 8.23E-05 |
| dihydroorotic acid | 0.000576 |
| 1,2-propanediol | 0.003702 |
| 2-isopropylmalate | 8.23E-05 |
| alanine | 8.23E-05 |
| alpha-glycerolphosphorylcholine | 8.23E-05 |
| alpha-rhamnose | 8.23E-05 |
| arginine | 8.23E-05 |
| fumarate | 8.23E-05 |
| glc+glc-6P | 0.000329 |
| hypoxanthine | 8.23E-05 |
| inosine | 8.23E-05 |
| isoleucine | 0.001563 |
| lysine | 8.23E-05 |
| NAc-glutamate | 0.003702 |
| SAH | 8.23E-05 |
| SAM | 8.23E-05 |
| serine | 8.23E-05 |
| succinate | 8.23E-05 |
| threonine | 8.23E-05 |
| tryptophan | 8.23E-05 |
| UDP-glcA | 0.003702 |
| uridine | 0.000329 |
