# Supplementary material for: Stitching together Multiple Data Dimensions Reveals Interacting Metabolomic and Transcriptomic Networks That Modulate Cell Regulation
Source: PLoS Biol. 2012 Apr 3;10(4):e1001301. doi: 10.1371/journal.pbio.1001301 (PMC3317911; doi:10.1371/journal.pbio.1001301)
Supplement: Table S4 — Concentrations of 25 metabolites are different (t-test p<0.05) between VPS9 knockout and wild-type strains. (DOCX) [file pbio.1001301.s017.docx]

**Table S4.** Concentrations of 25 metabolites are different (t-test p-value<0.05) between *VPS9* knockout and wild type strains.

| **Metabolite** | **p-value** |
| --- | --- |
| 2-isopropylmalate | 0.00684192 |
| ADP | 0.0429404 |
| alanine | 0.0132491 |
| alpha-glycerolphosphorylcholine | 0.0481803 |
| alpha-rhamnose | 0.0196893 |
| ATP | 0.0156644 |
| beta-mannose | 0.0100102 |
| galactose-1P | 0.00559565 |
| glc+glc-6P | 0.032494 |
| glutamine | 0.0290261 |
| hypoxanthine | 0.00485671 |
| inosine | 0.00645152 |
| isobutyrate | 0.00683991 |
| isoleucine | 0.000132688 |
| leucine | 0.00266534 |
| lysine | 0.0271286 |
| NAD | 0.0353643 |
| serine | 0.0450876 |
| succinate | 0.0169976 |
| thiaminemonophosphate | 0.0434224 |
| threonine | 0.0470553 |
| trehalose | 0.0024283 |
| tryptophan | 0.0166901 |
| uracil | 0.0330266 |
| valine | 0.0460296 |
