# Supplementary material for: Stitching together Multiple Data Dimensions Reveals Interacting Metabolomic and Transcriptomic Networks That Modulate Cell Regulation
Source: PLoS Biol. 2012 Apr 3;10(4):e1001301. doi: 10.1371/journal.pbio.1001301 (PMC3317911; doi:10.1371/journal.pbio.1001301)
Supplement: Table S5 — Expression levels of eight genes are different (t-test p<0.01) between VPS9 knockout and wild-type strains. (DOCX) [file pbio.1001301.s018.docx]

**Table S5.** Expression levels of 8 genes are different (t-test p-value<0.01) between *VPS9* knockout and wild type strains.

| **Gene** | **p-value** | **log2 (fold change)** |
| --- | --- | --- |
| GCV3 | 0.000012 | 0.475377 |
| ILV5 | 0.000013 | 1.87198 |
| CHA1 | 0.000248 | -0.846073 |
| BAT2 | 0.000324 | 1.010828 |
| ADE17 | 0.000432 | 0.804962 |
| YHR162W | 0.000598 | 1.000147 |
| GCV2 | 0.004854 | 0.496711 |
| ADE4 | 0.005773 | 0.576051 |
