# Supplementary material for: Stitching together Multiple Data Dimensions Reveals Interacting Metabolomic and Transcriptomic Networks That Modulate Cell Regulation
Source: PLoS Biol. 2012 Apr 3;10(4):e1001301. doi: 10.1371/journal.pbio.1001301 (PMC3317911; doi:10.1371/journal.pbio.1001301)
Supplement: Table S6 — Primers used for real-time PCR quantification of Chromosome XIII hotspot subnetwork genes. (DOCX) [file pbio.1001301.s019.docx]

**Table S6.** Primers used for real-time PCR quantification of chromosome XIII hotspot subnetwork genes.

| Primer id | sequence | Tm | product size | gene id/comment |
| --- | --- | --- | --- | --- |
| PSB148F | TCGATGTTGCCAAGAGATTG | ~60 | 211 | GCV2 |
| PSB148R | GAATGAGGGGCATTTTTCAA | ~60 | 211 | GCV2 |
| PSB149F | CGAATACGCTCGCAAATACA | ~60 | 177 | CHA1 |
| PSB149R | GCTAATTTTGAGCCCAGTGC | ~60 | 177 | CHA1 |
| PSB150F | CGTTGAACCACCAAAGGACT | ~60 | 139 | ILV5 |
| PSB150R | GTGAGCCTTACCGGTGACAT | ~60 | 139 | ILV5 |
| PSB151F | CCCGTTCAGGAACCACTCTA | ~60 | 180 | MCT1 |
| PSB151R | CATGGTGAATTGCACAGTCC | ~60 | 180 | MCT1 |
| PSB152F | GCAGTCTTCAGGCTGCTCTT | ~60 | 179 | VPS9 |
| PSB152R | GCCGTTTGGCTGTAACTCAT | ~60 | 179 | VPS9 |
| PSB153F | GGCCAAATTCCAACAGAAGA | ~60 | 203 | ADE17 |
| PSB153R | GATGGAGCGGCAATGTATTT | ~60 | 203 | ADE17 |
| PSB154F | TGTTGAGTTGCCAGAAGTGG | ~60 | 213 | GCV3 |
| PSB154R | ACATTAACGCCCTCACCAAG | ~60 | 213 | GCV3 |
| PSB155F | GAGAAAGCGGTTGAGTTTGC | ~60 | 219 | GCV1 |
| PSB155R | GGCATAGCCATTGAACCAGT | ~60 | 219 | GCV1 |
| PSB156F | TGCATTTCTGGGCTCCTACT | ~60 | 182 | YHR162W |
| PSB156R | CGAGTTGACAGAAGCCAACA | ~60 | 182 | YHR162W |
| PSB157F | ATAGCCTGGACGAGGAGACA | ~60 | 168 | PCL7 |
| PSB157R | CTTCGGTCTCATCCGACACT | ~60 | 168 | PCL7 |
| PSB158F | TCTGAGAAGGCCGAACAAGT | ~60 | 188 | MMF1 |
| PSB158R | GAAGCAACACCAACACAGGA | ~60 | 188 | MMF1 |
| PSB159F | AAAAACCTCAGGGTGCAATG | ~60 | 195 | ALT2 |
| PSB159R | AAAACGTTGTTCGCACATGA | ~60 | 195 | ALT2 |
| PSB160F | AAAACGGGCAAGAAGGAACT | ~60 | 211 | BAT2 |
| PSB160R | CAATCGCAGCAGTACCAGAA | ~60 | 211 | BAT2 |
| PSB161F | GTTGTTATTCCCGTCCCAGA | ~60 | 141 | ADE4 |
| PSB161R | AACCCTTTCCCTCTGGTTTG | ~60 | 141 | ADE4 |
| PSB162F | ATTCAGGTTTCCCACAGCAG | ~60 | 196 | UBA4 |
| PSB162R | TGAAATGCGTCCACACTGAT | ~60 | 196 | UBA4 |
| PSB163F | CAATACCATCCCGAAAATGG | ~60 | 229 | ZRT3 |
| PSB163R | TCATTGCAAATCCTTCGACA | ~60 | 229 | ZRT3 |
| PSB164F | TTCCAACGGTACCGATTCTC | ~60 | 149 | SHM2 |
| PSB164R | CACCTGGAACCAAAGCAGAT | ~60 | 149 | SHM2 |
| PSB167F | CTGCCGGTATTGACCAAACT | ~60 | 144 | ACT1 |
| PSB167R | CGGTGATTTCCTTTTGCATT | ~60 | 144 | ACT1 |
