# Supplementary material for: Stitching together Multiple Data Dimensions Reveals Interacting Metabolomic and Transcriptomic Networks That Modulate Cell Regulation
Source: PLoS Biol. 2012 Apr 3;10(4):e1001301. doi: 10.1371/journal.pbio.1001301 (PMC3317911; doi:10.1371/journal.pbio.1001301)
Supplement: Table S7 — Abundances of 27 metabolites are different (t-test p<0.05) between PHM7 knockout and wild-type strains. (DOCX) [file pbio.1001301.s020.docx]

**Table S7.** Abundances of 27 metabolites are different (t-test p-value<0.05) between *PHM7* knockout and wild type strains.

| **Metabolite Name** | **p-value** | **averaged fold change** |
| --- | --- | --- |
| Acetyl-CoA/CoA | 0.037116 | 1.295655 |
| ADP | 0.036269 | 1.593625 |
| alanine | 0.028949 | 1.509545 |
| alpha-glycerolphosphorylcholine | 0.020539 | 1.53016 |
| alpha-rhamnose | 0.049984 | 1.380973 |
| arginine | 0.035091 | 1.396791 |
| asparagine | 0.026866 | 1.424905 |
| beta-mannose | 0.014058 | 1.55396 |
| formate | 0.043025 | 1.415889 |
| glutamate | 0.021147 | 1.376064 |
| glutamine | 0.022404 | 1.40395 |
| glutathioine | 0.002056 | 1.43414 |
| hypoxanthine | 0.019693 | 1.436214 |
| inosine | 0.023312 | 1.242588 |
| isoleucine | 0.036928 | 1.446695 |
| leucine | 0.033039 | 1.393054 |
| lysine | 0.032729 | 1.44386 |
| NAD | 0.044397 | 1.376574 |
| niacinamide | 0.007493 | 1.554312 |
| phosphoenolpyruvate | 0.031787 | 1.824876 |
| pyroglutamate | 0.025486 | 1.599264 |
| serine | 0.027924 | 1.46978 |
| threonine | 0.047052 | 1.430648 |
| trehalose | 0.030625 | 2.40586 |
| tyrosine | 0.023863 | 1.44858 |
| uridine | 0.040121 | 1.330387 |
| valine | 0.028797 | 1.488951 |
