# Supplementary material for: Stitching together Multiple Data Dimensions Reveals Interacting Metabolomic and Transcriptomic Networks That Modulate Cell Regulation
Source: PLoS Biol. 2012 Apr 3;10(4):e1001301. doi: 10.1371/journal.pbio.1001301 (PMC3317911; doi:10.1371/journal.pbio.1001301)
Supplement: Table S8 — Composition of the synthetic complete medium. (DOCX) [file pbio.1001301.s021.docx]

**Table S8.** Composition of the synthetic complete medium.

| **Nutrient** | **Concentration** | **Concentration (g/L)** | **MW (g/mole)** | **mM in Medium** |
| --- | --- | --- | --- | --- |
| **Amino Acids** |  |  |  |  |
| Methionine | 20 mg/L | 2.0E-02 | 149.2 | 1.3E-01 |
| Tyrosine | 60 mg/L | 6.0E-02 | 181.2 | 3.3E-01 |
| Isoleucine | 80 mg/L | 8.0E-02 | 131.2 | 6.1E-01 |
| Phenylalanine | 50 mg/L | 5.0E-02 | 165.2 | 3.0E-01 |
| Glutamate | 100 mg/L | 1.0E-01 | 147.1 | 6.8E-01 |
| Threonine | 200 mg/L | 2.0E-01 | 119.1 | 1.7E+00 |
| Aspartate | 100 mg/L | 1.0E-01 | 133.1 | 7.5E-01 |
| Valine | 150 mg/L | 1.5E-01 | 117.2 | 1.3E+00 |
| Serine | 400 mg/L | 4.0E-01 | 105.1 | 3.8E+00 |
| Arginine.HCl | 20 mg/L | 2.0E-02 | 210.7 | 9.5E-02 |
| Histidine | 20 mg/L | 2.0E-02 | 155.2 | 1.3E-01 |
| Leucine | 80 mg/L | 8.0E-02 | 131.2 | 6.1E-01 |
| Lysine.2HCl | 120 mg/L | 1.2E-01 | 219.1 | 5.5E-01 |
| Tryptophan | 80 mg/L | 8.0E-02 | 204.2 | 3.9E-01 |
|  |  |  |  |  |
| **Nitrogenous Bases and Carbon Source** |  |  |  |  |
| Adenine | 20 mg/L | 2.0E-02 | 135.1 | 1.5E-01 |
| Uracil | 35 mg/L | 3.5E-02 | 112.1 | 3.1E-01 |
| Glucose | 20 g/L | 2.0E-02 | 180.2 | 1.1E-01 |
|  |  |  |  |  |
| **Yeast Nitrogen Base** |  |  |  |  |
| Biotin | 2 µg/L | 2.0E-06 | 244.3 | 8.2E-06 |
| Calcium Pantothenate | 400 µg/L | 4.0E-04 | 238.3 | 1.7E-03 |
| Folic acid | 2 µg/L | 2.0E-06 | 441.4 | 4.5E-06 |
| Niacin | 400 µg/L | 4.0E-04 | 123.1 | 3.2E-03 |
| p-Aminobenzoic acid | 200 µg/L | 2.0E-04 | 137.1 | 1.5E-03 |
| Pyridixine HCl | 400 µg/L | 4.0E-04 | 205.6 | 1.9E-03 |
| Riboflavin | 200 µg/L | 2.0E-04 | 376.4 | 5.3E-04 |
| Thiamine HCl | 400 µg/L | 4.0E-04 | 337.3 | 1.2E-03 |
| Inositol | 2 mg/L | 2.0E-03 | 180.2 | 1.1E-02 |
| Boric acid | 500 µg/L | 5.0E-04 | 61.8 | 8.1E-03 |
| Copper sulfate | 40 µg/L | 4.0E-05 | 159.6 | 2.5E-04 |
| Potassium iodide | 100 µg/L | 1.0E-04 | 166.0 | 6.0E-04 |
| Ferric chloride | 200 µg/L | 2.0E-04 | 162.2 | 1.2E-03 |
| Manganese sulfate.H2O | 400 µg/L | 4.0E-04 | 169.0 | 2.4E-03 |
| Sodium molybdate | 200 µg/L | 2.0E-04 | 205.9 | 9.7E-04 |
| Zinc sulfate.H2O | 400 µg/L | 4.0E-04 | 179.5 | 2.2E-03 |
| Potassium phosphate monobasic | 1 g/L | 1.0E+00 | 136.1 | 7.3E+00 |
| Magnesium sulfate | 0.5 g/L | 5.0E-01 | 120.4 | 4.2E+00 |
| Sodium chloride | 0.1 g/L | 1.0E-01 | 58.4 | 1.7E+00 |
| Calcium chloride | 0.1 g/L | 1.0E-01 | 111.0 | 9.0E-01 |
