# Supplementary material for: Stitching together Multiple Data Dimensions Reveals Interacting Metabolomic and Transcriptomic Networks That Modulate Cell Regulation
Source: PLoS Biol. 2012 Apr 3;10(4):e1001301. doi: 10.1371/journal.pbio.1001301 (PMC3317911; doi:10.1371/journal.pbio.1001301)
Supplement: Table S9 — Correlation coefficients of all pairs of nodes in the URA3 subnetwork. (DOCX) [file pbio.1001301.s022.docx]

**Table S9** Correlation coefficients of all pairs of nodes in the URA3 subnetwork.
