# Supplementary material for: Stitching together Multiple Data Dimensions Reveals Interacting Metabolomic and Transcriptomic Networks That Modulate Cell Regulation
Source: PLoS Biol. 2012 Apr 3;10(4):e1001301. doi: 10.1371/journal.pbio.1001301 (PMC3317911; doi:10.1371/journal.pbio.1001301)
Supplement: Table S11 — Causality test results for the 18 nodes in the URA3 network. (DOCX) [file pbio.1001301.s024.docx]

**Table S11.** Causality test results for the 18 nodes in the URA3 network. Rows represent causal parents and columns represent reactive children. The relationships in green are causal relationships. For example, the relationship URA3 🡪 YEL016C is green. It means URA3 has been statistically inferred as causal for YEL016C expression, and the corresponding link URA3 🡪 YEL016C is possible in the network. Red relationships represent impossible relationships. Red cells may be the result of causal/reactive calls (such as YEL016C 🡪 URA3) or the result of independent calls (such as URA4 🡪 AQR1). Relationships in yellow represent no call or not tested so that both directions are possible.
