# Supplementary material for: Stitching together Multiple Data Dimensions Reveals Interacting Metabolomic and Transcriptomic Networks That Modulate Cell Regulation
Source: PLoS Biol. 2012 Apr 3;10(4):e1001301. doi: 10.1371/journal.pbio.1001301 (PMC3317911; doi:10.1371/journal.pbio.1001301)
Supplement: Text S1 — An example in constructing BNs to make novel discoveries. (DOC) [file pbio.1001301.s025.doc]

**Text S1**

**An example in constructing Bayesian networks to make novel discoveries**

To illustrate the steps in our Bayesian network reconstruction procedure and to examine contributions from different data types, we focus on genes and metabolites involved in de novo biosynthesis of pyrimidine ribonucleotides as shown in Figure 4C. For simplicity we focus the reconstruction on this smaller subset of genes, but note that there are differences between reconstructing networks using only a subset of nodes and reconstructing networks using all nodes and extracting subnetworks from the full network. Unlike association-based networks (e.g., coexpression networks) that consider pairwise relationships, the Bayesian network reconstruction process evaluates relationships of all pairs of nodes by conditioning on all other nodes. Therefore, there may be minor differences in subnetworks extracted from networks reconstructed from all nodes and networks constructed from the subset of nodes comprising the subnetwork of interest.

*Step 1: Identification of the URA3 centered de novo biosynthesis of pyrimidine ribonucleotides subnetwork.* There are 18 nodes in the subnetwork showed in Figure 4C in the main text. These nodes are highly correlated (see Table S9 for the correlation coefficient matrix), with 68% of all pairwise relationship significant at the 0.01 significance level. The continuous gene expression data for these 18 genes were discretized into three states representing down-regulated, no-change, and up-regulated states, and then the mutual information of all pairs of nodes was calculated (Table S10). 54% of all pairs are significant at p-value <0.01 (mutual information of permutated data was calculated and fit into a normal distribution, which was then used to assess significance of mutual information of real data). All of the trait values corresponding to these nodes are significantly associated with the genotypes at the URA3 locus (Figure S9).

*Step 2: Reconstructing networks using only expression and metabolite traits (excluding DNA variation data).* The process of reconstructing networks using only trait data is straight-forward (Figure S10). The trait data is input into a standard Bayesian network reconstruction program in which 1,000 network structures are generated from a Monte Carlo Markov Chain (MCMC) process using different random seed numbers (1000 random seed numbers were generated by a master process, then each slave process started a MCMC process using one of generated seed numbers). Once the 1,000 network structures have been generated, common features are extracted to derive a consensus network. With this construction, the consensus network may contain loops, which are prohibited in Bayesian networks. Therefore, to ensure the consensus network structure is a directed acyclic graph, the edges in the original consensus network are removed if and only if 1) the edge was involved in a loop, and 2) the edge was the most weakly supported of all edges making up the loop. The network resulting from this process is shown in Figure S11. The root node of the Bayesian network is MCM1.

*Step 3: Constructing priors using eQTL data.* The network in step 2 is constructed without considering any of the genetic data. Because eQTL data represent a systematic source of perturbation on the expression data, integrating these data have the potential to better resolve causal relationships. Towards this end, expression and genotype data were compared to detect eQTLs. As shown in Figure S9, nearly all of the nodes have QTLs linked to a single locus on chromosome V. Expression traits that associate with a common eQTL can be subjected to a statistical test to infer causal relationships between the traits, as previously described. The causality test results for the 18 nodes are shown in Table S11. Among the nodes tested, URA3 and YEL016C have cis-acting eQTLs linked to the locus. Nodes with cis-acting eQTLs are allowed to be causal parent nodes to nodes with trans-acting QTLs. However, nodes with trans-QTLs are not allowed to be causal parent nodes to nodes with cis-acting eQTLs. The relationships highlighted in red are not possible as determined by the causality test results. As a result, these relationships are not searched as part of the network reconstruction process.

*Step 4: Constructing priors using KEGG data.* The network constructed in step 2 also does not consider known relationships among genes and metabolites as defined by canonical pathways. The relationships between enzymes and metabolites are well established in many cases. To incorporate this knowledge into the network reconstruction process, we construct priors using canonical pathway data in the following way. There are two metabolites in the *URA3* subnetwork. Their distances to each other and related enzymes as defined in the KEGG database are:

| Metabolite 1 | Metabolite 2 | Distance in KEGG Database |
| --- | --- | --- |
| Orotic acid | Dihydroorotic acid | 1 |
| Orotic acid | URA1 | 1 |
| Orotic acid | URA3 | 2 |
| Orotic acid | URA4 | 2 |
| Orotic acid | URA2 | 3 |
| Dihydroorotic acid | URA1 | 1 |
| Dihydroorotic acid | URA4 | 1 |
| Dihydroorotic acid | URA3 | 3 |
| Dihydroorotic acid | URA2 | 2 |

We construct the structure prior for the gene expression of an enzyme $e$ affecting a metabolite concentration using their shortest distance $d_{m,e}$ as $p(m->e)\propto e^{-\lambda d_{m,e}}$. The shorter the distance, the stronger the prior. In the 18-node subnetwork, the prior for two nodes connected by chance is 0.0588 (=1/17). The structure priors for the above metabolite/enzyme relationships are:

| Metabolite 1 | Metabolite 2 | Prior Probability |
| --- | --- | --- |
| Dihydroorotic acid | Orotic acid | 0.367879 |
| Orotic acid | URA1 | 0.367879 |
| URA1 | Orotic acid | 0.367879 |
| Orotic acid | URA3 | 0.135335 |
| URA3 | Orotic acid | 0.135335 |
| Orotic acid | URA4 | 0.135335 |
| URA4 | Orotic acid | 0.135335 |
| Dihydroorotic acid | URA1 | 0.367879 |
| URA1 | Dihydroorotic Acid | 0.367879 |
| Dihydroorotic acid | URA4 | 0.367879 |
| URA4 | Dihydroorotic acid | 0.367879 |
| Dihydroorotic acid | URA2 | 0.135335 |
| URA2 | Dihydroorotic acid | 0.135335 |

*Step 5: Constructing networks using expression data, metabolite data, and the genetic and canonical pathway priors defined in Steps 3 and 4.* The process of reconstructing networks using trait data and priors from other data types is similar to the reconstruction process applied to trait data only described in *Step 2*  (Figure S12). In addition to trait data, priors derived from other data types are also input into a standard Bayesian network reconstruction process as shown in Figure S10. The trait data of the 18 nodes and related priors were input into the network reconstruction process, and the resulting network is showed in Figure S13. The root node of the Bayesian network is URA3, which is the gene with the cis-acting eQTL associated with other traits in the network.

*Step 6: Comparing the networks constructed in Steps 2 and 5.* The main difference between the networks depicted in Figures S11 and S13 are the head nodes. In general, directed links in a Bayesian network do not necessarily represent a causal relationship. The network constructed from the trait data only reflects relationships not supported by the genetic perturbation data, but that are well captured by the more integrated network described in Step 5. For example, the link RIB4 🡪 URA3 depicted in Figure S11 is opposite that identified in Figure S5. Because the genetic perturbation at the URA3 locus affects the expression activity of that gene in cis and the expression activity of the gene RIB4 in trans, the experimentally supported relationship is URA3 🡪 RIB4. We note that the enzyme/metabolite and metabolite/metabolite relationships are similar with or without the priors derived from the KEGG pathways.

All data and software used to construct the Bayesian networks for this example and in the main text are available at <http://www.mssm.edu/research/institutes/genomics-institute/rimbanet>.

**Text S1 References**

1. Millstein J, Zhang B, Zhu J, Schadt EE (2009) Disentangling molecular relationships with a causal inference test. BMC Genet 10: 23.

2. Schadt EE, Lamb J, Yang X, Zhu J, Edwards S, et al. (2005) An integrative genomics approach to infer causal associations between gene expression and disease. Nat Genet 37: 710-717.

3. Doss S, Schadt EE, Drake TA, Lusis AJ (2005) Cis-acting expression quantitative trait loci in mice. Genome Res 15: 681-691.

4. Zhu J, Lum PY, Lamb J, GuhaThakurta D, Edwards SW, et al. (2004) An integrative genomics approach to the reconstruction of gene networks in segregating populations. Cytogenet Genome Res 105: 363-374.
